# A myeloid leukemia factor homolog is involved in tolerance to stresses and stress-induced protein metabolism in *Giardia lamblia*

**DOI:** 10.1186/s13062-023-00378-6

**Published:** 2023-04-24

**Authors:** Jui-Hsuan Wu, Jen-Chi Lee, Chun-Che Ho, Pei-Wei Chiu, Chin-Hung Sun

**Affiliations:** Department of Tropical Medicine and Parasitology, College of Medicine, National Taiwan University, Taipei, 100 Taiwan, Republic of China

**Keywords:** Cyst, MLF, CDK2 mutant, *Giardia*, Differentiation, Nocodazole, DTT, G418, FYVE, ATG8-like

## Abstract

**Background:**

The eukaryotic membrane vesicles contain specific sets of proteins that determine vesicle function and shuttle with specific destination. *Giardia lamblia* contains unknown cytosolic vesicles that are related to the identification of a homolog of human myeloid leukemia factor (MLF) named MLF vesicles (MLFVs). Previous studies suggest that MLF also colocalized with two autophagy machineries, FYVE and ATG8-like protein, and that MLFVs are stress-induced compartments for substrates of the proteasome or autophagy in response to rapamycin, MG132, and chloroquine treatment. A mutant protein of cyclin-dependent kinase 2, CDK2m3, was used to understand whether the aberrant proteins are targeted to degradative compratments. Interestingly, MLF was upregulated by CDK2m3 and they both colocalized within the same vesicles. Autophagy is a self-digestion process that is activated to remove damaged proteins for preventing cell death in response to various stresses. Because of the absence of some autophagy machineries, the mechanism of autophagy is unclear in *G. lamblia*.

**Results:**

In this study, we tested the six autophagosome and stress inducers in mammalian cells, including MG132, rapamycin, chloroquine, nocodazole, DTT, and G418, and found that their treatment increased reactive oxygen species production and vesicle number and level of MLF, FYVE, and ATG8-like protein in *G. lamblia*. Five stress inducers also increased the CDK2m3 protein levels and vesicles. Using stress inducers and knockdown system for MLF, we identified that stress induction of CDK2m3 was positively regulated by MLF. An autophagosome-reducing agent, 3-methyl adenine, can reduce MLF and CDK2m3 vesicles and proteins. In addition, knockdown of MLF with CRISPR/Cas9 system reduced cell survival upon treatment with stress inducers. Our newly developed complementation system for CRISPR/Cas9 indicated that complementation of MLF restored cell survival in response to stress inducers. Furthermore, human MLF2, like *Giardia* MLF, can increase cyst wall protein expression and cyst formation in *G. lamblia*, and it can colocalize with MLFVs and interact with MLF.

**Conclusions:**

Our results suggest that MLF family proteins are functionally conserved in evolution. Our results also suggest an important role of MLF in survival in stress conditions and that MLFVs share similar stress-induced characteristics with autophagy compartments.

**Supplementary Information:**

The online version contains supplementary material available at 10.1186/s13062-023-00378-6.

## Background

*Giardia lamblia* causes outbreaks of waterborne diarrhea disease known as giardiasis that predominately afflicts developing countries [1–3]. It is transmitted by ingestion of cysts with a resistant extracellular wall from contaminated water or food [4]. Various animals are believed to be reservoirs for human infection, making it difficult to eradicate [4]. Following acute giardiasis with gastrointestinal symptoms, quality of life of patients may be affected by irritable bowel syndrome [5]. Antigenic variation has been proposed as a reason for chronic infection that is always found in children and may lead to malabsorption and childhood mortality [6].

Many protozoan pathogens of medical importance have a cyst form [7]. *G. lamblia* is a unique model for differentiation of protozoan pathogens, as its two life-cycle stages can be reproduced in vitro [1, 8]. Trophozoite is the form causing giardiasis, containing four pairs of flagella for movement in small intestine [4]. Cyst, the infective form, has structures derived from two trophozoites inside a thick wall [4, 9]. Encystation of trophozoites occurs in response to a different environment toward lower intestine [1, 2]. Synthesis and secretion of some special components, such as three cyst wall proteins (CWPs), are important for the assembly of a protective cyst wall [10–12]. During encystation, *cwp1-3* genes are up-regulated with similar kinetics by transcription factors GARP1, ARID1, MYB2, WRKY, PAX1, E2F1, and MBF1 [13–19].

*G. lamblia* raises great biological interest for understanding eukaryotic evolution, because it has many primitive features, including the utilization of bacteria-like anaerobic metabolism and the lack of clear homologs of many known basal transcription factors, DNA replication proteins, and RNA processing factors [2, 4, 20, 21]. It is possible that these factors do not exist or they are too divergent in *G. lamblia* [2, 21]. Only a few candidate proteins with similarity to proteasome subunits in yeast were identified from *G. lamblia* [22]. In addition, only several putative autophagy-related factors, including TOR, S6K1, PI3K, ATG1, ATG16, ATG18, ATG7, and ATG8, have been found from the *G. lamblia* genome database [23, 24]. These studies suggest that *G. lamblia* has a partial or unusual proteasome and autophagy pathway for protein metabolism [21, 23, 24].

Eukaryotic cells use autophagy to survive in various stresses, including starvation, oxidative stress, aging, and diseases [25–27]. Autophagy is a self-digestion process that facilitates the removal of misfolded proteins and damaged organelles and provides materials to synthesize the new proteins [25–27]. It is activated to remove oxidatively damaged proteins for preventing cell death in response to various stressful stimuli [25–27]. Autophagy is a normal degradation pathway, although the autophagic structures are not easily observed due to their transient character [28]. Autophagic structures can be observed in an easier way through increasing biogenesis and reducing degradation [28]. From research with mammals and yeast, various agents are known to cause oxidative stress conditions for reactive oxygen species (ROS) production that leads to stimulate autophagy [27]. These findings suggest that autophagic structures are stress-induced compartments and that autophagy is a protective mechanism in response to stress conditions [29].

Proteasome and autophagy systems involving in clearance of misfolded proteins, plays a positive role in differentiation of various cell types, such as neuronal, myocardial, and lens cells [30–33]. During ciliate encystment, autophagic activity increases to degrade many components, such as mitochondria, cilia, and some nuclear apparatus [34]. Growing evidence supports the presence of autophagy phenomenon in *G. lamblia* and other protozoa, including *Plasmodium, Trypanosoma*, and *Trichomonas* [23, 24, 35–39]. Treatment of metronidazole analogues results in formation of autophagic vacuoles with concentric membranes in *G. lamblia* [35].

As has been found in mammals and *Drosophila*, the myeloid leukemia factor (*mlf*) gene products are implicated in the regulation of cell differentiation [40, 41] To date, no MLF family has been identified in yeast, plants, or most protozoa, except only one MLF-like protein identified in *Giardia* [40–43]. Therefore, it is of interest to know the role or MLF in this protozoan parasite. Little is known about the precise function of MLFs. One possible MLF function is the role in regulation of unfolded and aggregated protein by interacting with a chaperone to maintain protein stability or to reduce the toxic effect protein aggregates in mammalian cells [44, 45]. Like mammalian MLF proteins that play critical roles in cell differentiation, we also found that *Giardia* MLF is a positive driver for encystation using a CRISPR/Cas9 system [42]. To study the regulation of protein degradation in *G. lamblia*, we have used a mutant with a deletion of kinase domain, cyclin-dependent kinase 2 mutant 3 (CDK2m3), and double staining for visualization of potential factors with a role in protein metabolism pathway [46, 47]. We found that MLF interacted and colocalized with Cdk2m3 in intracellular vesicles whose number are increased by MG132 and chloroquine treatment in *G. lamblia*, suggesting that MLF is important for aberrant protein substrates of the proteasome or autophagy [47]. MLF localized to some unknown high-speed membrane vesicles named MLF vesicles (MLFVs), that are not mitosomes or encystation-specific vesicles, but are related with degradative pathway for CDK2m3 aberrant protein [47–51]. MLF also interacted and colocalized with two autophagy markers, a FYVE protein with a conserved FYVE domain, which is like the autophagy marker ALFY, and an ATG8-like (ATG8L) protein [47]. Interestingly, FYVE was also largely co-localized with the lysosomal compartment (peripheral vesicles) in our findings [47] and other other studies [52], suggesting that unfolded cargos laterly target to lysosomes. The induction of MLF, FYVE and ATG8L expression during encystation suggests that they are important for encystation [47]. Furthermore, we found that, like MLF, FYVE and ATG8L also can induce encystation by increasing CWP1 level and cyst formation [47]. The addition of proteasome or autophagy inhibitors, MG132, rapamycin, or chloroquine, increased the protein levels and the numbers of MLF, FYVE, and ATG8L vesicles, and inhibited the cyst formation [47]. The results suggest that these factors play a positive role in encystation and function in protein clearance pathway to remove unwanted trophozoite proteins, which is important for encystation.

In this study, we will use a MLF-based assay to test the effect of autophagy-inducing agents on protein metabolism. Little is known of the mechanism of autophagy in *G. lamblia* because of the lack of some autophagy machineries, such as p62 and ATG5 [23, 24]. To establish whether the aberrant proteins are targeted to degradative compratments, we expressed HA-tagged epitope tagged CDK2m3 and treated with the various autophagy related agents. MG132, rapamycin, chloroquine, nocodazole, DTT, and G418 are known to induce stress responses that lead to the accumulation of ROS and stimulate autophagosome formation in mammalian cells [53–64]. We found that treatment with most stress inducers led to an increase in the levels of CDK2m3, MLF, FYVE, and ATG8L proteins and the numbers of their vesicles, and ROS production in *G. lamblia*. However, treatment with an autophagy inhibitor, 3-methyl adenine (3-MA), resulted in a decrease in the levels of CDK2m3 and MLF proteins and the numbers of their vesicles. Further use of CRISPR/Cas9 system and complementation system suggest a positive role of MLF in survival from stress-induced cell death in the stress inducer treatment. We also found that MLF regulated stress-induced CDK2m3 formation, and that the human MLF2 had activity in inducting encystation, like *Giardia* MLF. Our results suggest that MLF is an evolutionarily conserved factor and functions in survival in stress and mutant protein metabolism, and that MLFVs, like autophagy compartments, have stress-induced characteristics and can be decreased by 3-MA.

## Results

### Human MLF2 is functionally similar to *Giardia* MLF

**Fig. 1:**
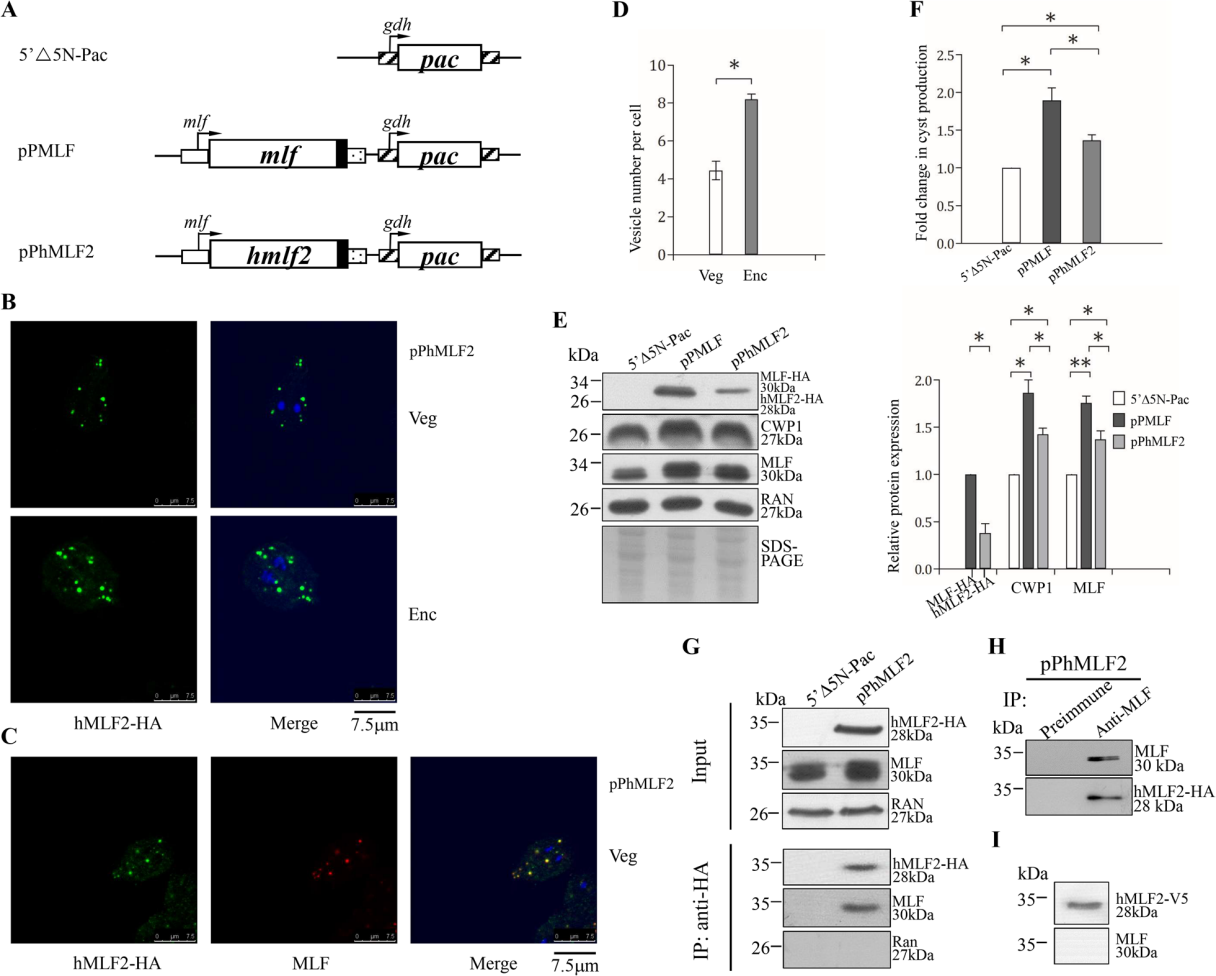
Colocalization and interaction of *Giardia* MLF and hMLF2. **A** Diagrams of the 5′5N-Pac, pPMLF and pPhMLF2 plasmids. The *pac* gene (open box) is under the control of the 5′- and 3′ -flanking regions of the *gdh* gene (striated box). In constructs pPMLF and pPhMLF2, the *mlf* and *hmlf2* genes are under the control of the 5′ -flanking region of the *mlf* gene (open box) and the 3′ -flanking region of the *ran* gene (dotted box). The filled black box indicates the coding sequence of the HA epitope tag. **B** Immunofluorescence analysis of hMLF2 distribution. The pPhMLF2 stable transfectants were cultured in growth (Veg, vegetative growth, upper panels) or encystation medium for 24 h (Enc, encystation, lower panels) and then subjected to immunofluorescence analysis using anti-HA antibody for detection. **C** Colocalization of MLF and hMLF2. The pPhMLF2 stable transfectants were cultured in growth (Veg, vegetative growth) and then subjected to immunofluorescence assay. The endogenous *Giardia* MLF protein and vector expressed HA-tagged hMLF2 protein were detected by anti-MLF and anti-HA antibodies, respectively. **D** Quantification of hMLF2 vesicles in pPhMLF2 cell line during vegetative and encysting stages using Imaris software. *, *p* < 0.05 (*n* = 200–300 cells/condition). **E** Expression of hMLF2 increased the CWP1 protein level. The 5’Δ5N-Pac, pPMLF, and pPhMLF2 stable transfectants were cultured in growth medium and then subjected to SDS-PAGE and Western blot analysis. The blot was probed with anti-HA, anti-CWP1, anti-MLF, and anti-RAN antibodies, respectively. Equal amounts of protein loading were confirmed by SDS-PAGE with Coomassie Blue staining. A similar level of the RAN protein was detected. The intensity of bands from three Western blot assays was quantified using Image J. The ratio of each target protein over the loading control RAN is calculated. Fold change is calculated as the ratio of the difference between pPMLF/pPhMLF2 cell line and the control cell line, to which a value of 1 was assigned. Results are expressed as mean ± 95% confidence intervals. *, *p* < 0.05. **, *p* < 0.01. **F** hMLF2 expression increased cyst formation. The 5’Δ5N-Pac, pPMLF, and pPhMLF2 stable transfectants were cultured in growth medium and then subjected to cyst count. The sum of total cysts is expressed as relative expression level over control. Values are shown as means ± 95% confidence intervals. *, *p* < 0.05. **G** Interaction between *Giardia* MLF and hMLF2. Expression of HA-tagged hMLF2 and MLF proteins was detected in whole cell extracts for co-immunoprecipitation assays (Input, upper panel). The 5’Δ5N-Pac and pPhMLF2 stable transfectants were cultured in growth medium and then subjected to SDS-PAGE and Western blot. The blot was probed with anti-HA, anti-MLF, and anti- RAN antibodies, respectively. Interaction between hMLF2 and MLF was detected by co-immunoprecipitation assays (bottom panel). The 5’Δ5N-Pac and pPhMLF2 stable transfectants were cultured in growth medium. Proteins from cell lysates were immunoprecipitated using anti-HA antibody conjugated to beads. The precipitates were analyzed by Western blotting with anti-HA, anti-MLF, and anti-RAN antibodies, respectively, as indicated. **H** Confirmation of interaction between hMLF2 and MLF. The pPhMLF2 stable transfectants were cultured in growth medium. Proteins from cell lysates were immunoprecipitated using anti-MLF antibody to assess binding of MLF to hMLF2. Preimmune serum was used as a negative control. The precipitates were analyzed by Western blotting with anti-MLF, and anti-HA antibodies, respectively, as indicated. **I** hMLF2 protein was not recognize by anti-MLF antibody. Purified V5-tagged hMLF2 protein was analyzed by Western blotting with anti-V5 and anti-MLF, respectively, as indicated

It has been shown that human MLF2 (hMLF2) may function in maintaining protein stability and vesicle trafficking [41, 65]. Like the role of human MLFs in cell differentiation, *Giardia* MLF also can induce encystation for the differentiation into cysts [40–42]. We tried to understand whether MLF function changes in evolution by testing the effect of hMLF2 expression in *G. lamblia* (Fig. 1A). Interestingly, hMLF2 had similar vesicle localization compared with the *Giardia* MLF in *G. lamblia* (Fig. 1B). In addition, we found a colocalization of hMLF2 and *Giardia* MLF and an increase in number of hMLF2 vesicles during encystation (Fig. 1C, D). Pearson correlation coefficient analysis was performed to quantitate the degree of colocalization of hMLF2 and *Giardia* MLF [66]. The average value of Pearson correlation coefficient was 0.92 (*n* = 12), indicating a high colocalization degree. There was a significant induction in cyst number and CWP1 protein level in the hMLF2- expressing cell lines compared with the *Giardia* MLF-expressing cell line (Fig. 1E, F). Since the protein level of hMLF2 significantly decreased as compared to that of *Giardia* MLF (Fig. 1E), the encystation-inducing activity of hMLF2 is possibly similar to that of *Giardia* MLF. Co-immunoprecipitation analysis also revealed that hMLF2 interacted with *Giardia* MLF in a complex (Fig. 1G, H). As a control, the anti-HA antibody did not immunoprecipitate RAN in the hMLF2-expressing cell line (Fig. 1G). The reciprocal immunoprecipitation also confirmed the interaction of hMLF2 and *Giardia* MLF (Fig. 1H). The hMLF2 protein cannot be recognized by our anti-MLF antibody which was used to reveal the specificity of MLF (Fig. 1I). The similar function of hMLF2 and *Giardia* MLF suggests that the members of the MLF family have been conserved during evolution.

### Mutation of basic region decreased MLF function

**Fig. 2:**
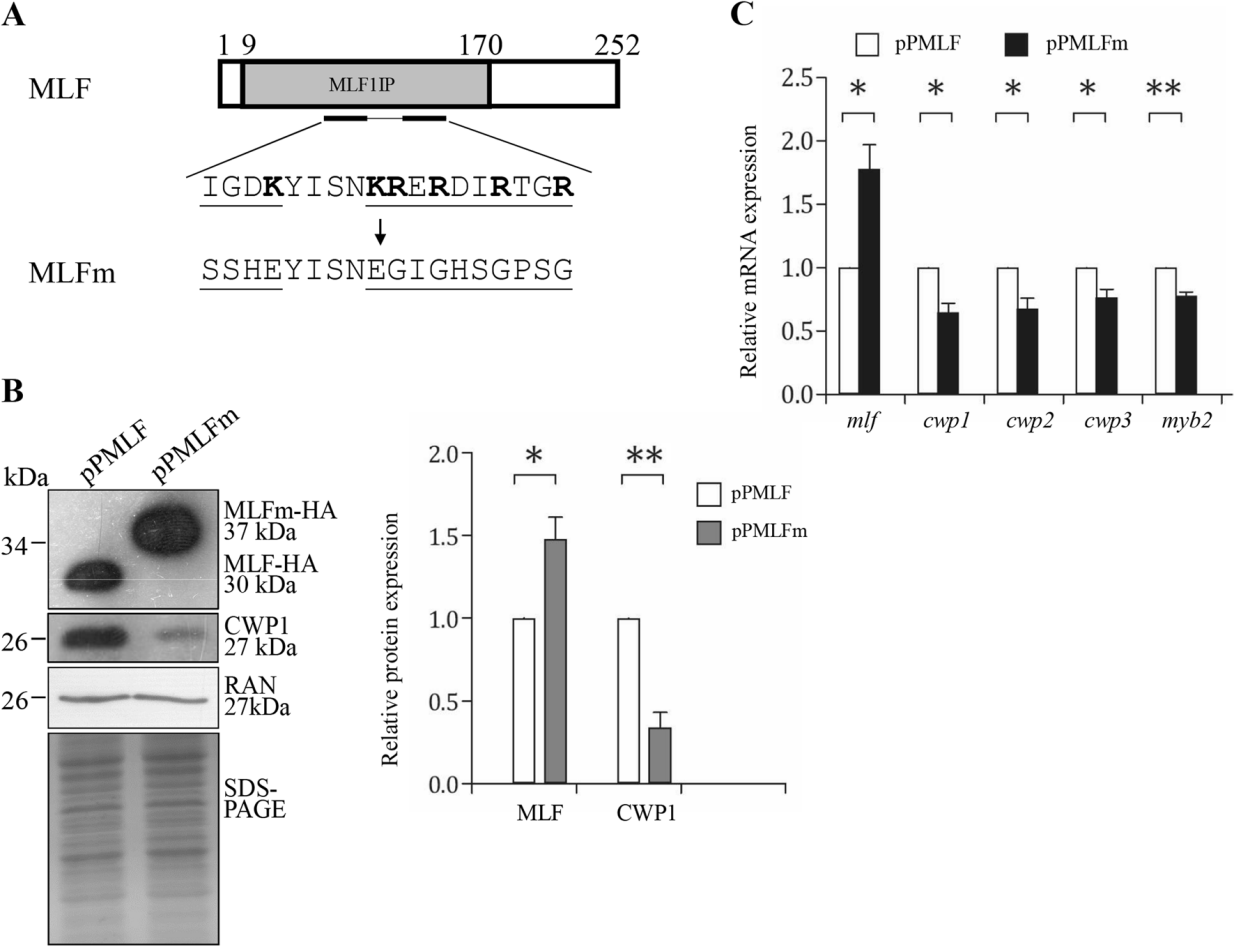
A MLF mutant with mutation residues 128–145 (enriched in basic amino acids, MLFm) showed decreased levels of the CWP1 protein and *cwp1-3* and *myb2* gene expression. **A** Diagrams of the MLF and MLFm proteins. The gray box indicates the Myelodysplasia-myeloid leukemia factor 1-interacting protein (MLF1IP) domain identified in pfam. MLFm contains mutations (underlined) of two stretches of sequences containing basic amino acids (bold) located in residues 128–145. The *mlf* gene was mutated and subcloned to replace the wild type *mlf* gene in the backbone of pPMLF (Fig. 1A), and the resulting plasmid pPMLFm was transfected into *G. lamblia*. **B** MLFm expression decreased the CWP1 level. The pPMLF and pPMLFm stable transfectants cultured in growth medium were subjected to Western blot analysis using anti-HA, anti-CWP1, and anti-Ran antibodies, respectively. The band intensity from triplicate Western blots was quantified using Image J as described in Fig. 1E. *, *p* < 0.05. **, *p* < 0.01. **C** Quantitative real-time PCR assays of transcript levels in the MLF and MLFm- expressing cell lines during vegetative growth. Real-time RT-PCR analysis was performed using primers specific for *mlf*, *cwp1*, *cwp2*, *cwp3*, *myb2*, and 18S ribosomal RNA genes, respectively. Similar levels of the 18S ribosomal RNA were detected. The mRNA levels were normalized to the 18S ribosomal RNA levels. The ratio of mRNA levels in the pPMLFm cell line to levels in the pPMLF cell line is shown and expressed as the mean ± 95% confidence intervals of at least three separate experiments. *, *p* < 0.05. **, *p* < 0.01

We also performed mutation analysis to understand the role of *Giardia* MLF. It has been shown that *Giardia* MLF can induce the expression of *cwp1-3* and *myb2* genes [42]. We mutated several residues inside a region enriched in basic amino acids to disrupt the protein stability (128 to 145, MLFm) (Fig. 2A). The basic amino acids, arginine and lysine, are important for protein stability due to formation of ionic interactions with their positive charges [67]. We found that the CWP1 level significantly decreased in the MLFm-expressing cell lines relative to the wild-type MLF-expressing cell line (Fig. 2B). The size of MLFm-HA is larger than that of MLF-HA, possibly due to the charge difference between MLFm and wild-type MLF. Migration of proteins can also be determined by their charges [68]. The mRNA levels of *mlf*, *cwp1-3* and *myb2* significantly decreased in the MLFm-expressing cell lines in comparison with the wild-type MLF-expressing cell line (Fig. 2C). The results suggest that MLFm displays reduced ability to induce encystation.

### Establishment of a CRISPR/Cas9 system with neomycin selection

**Fig. 3:**
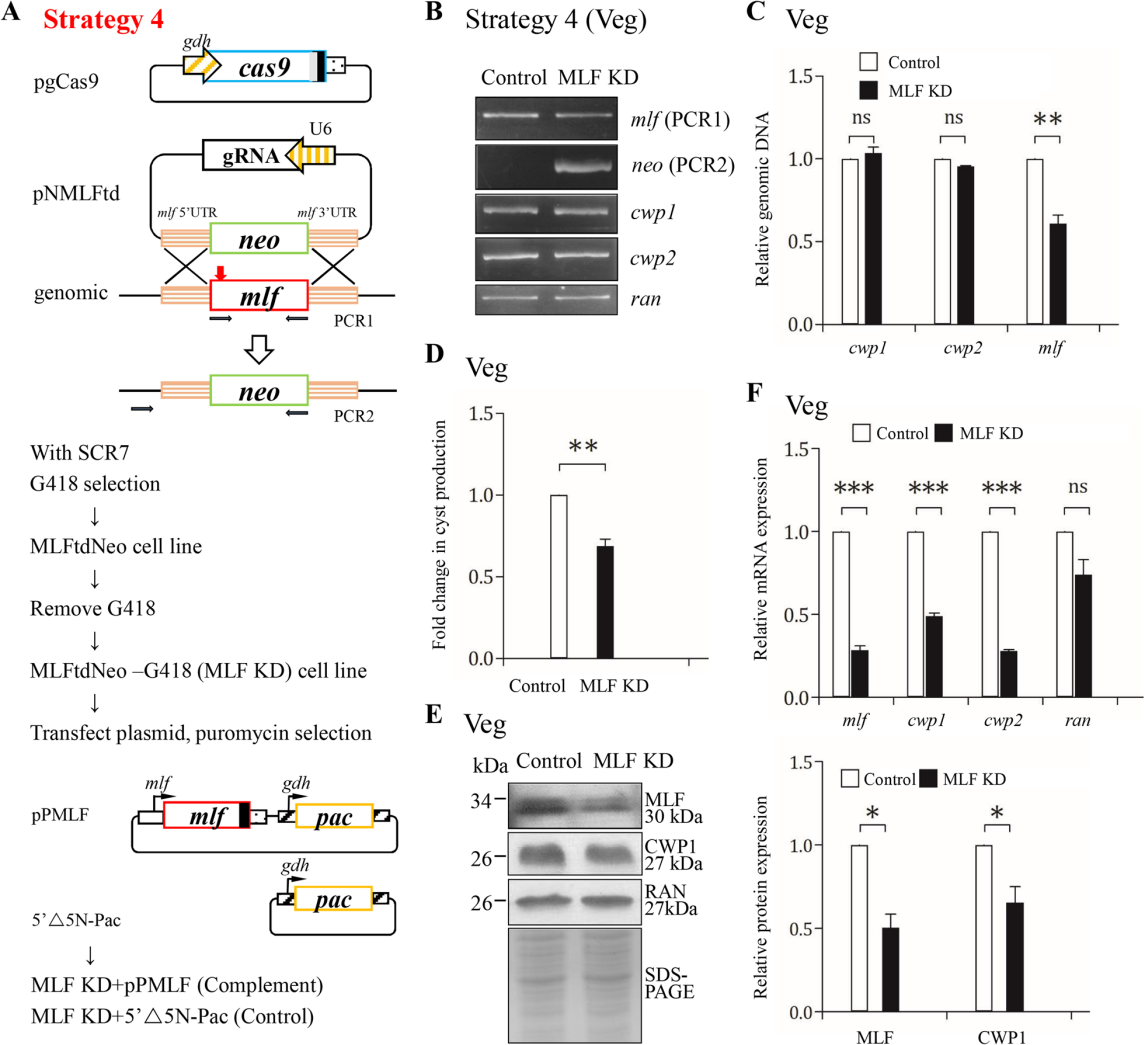
Targeted disruption of the *mlf* gene resulted in decreased expression of the *cwp1* and *cwp2* genes during vegetative growth using strategy 4. **A** Schematic presentation of the pgCas9 and pNMLFtd plasmids. In construct pgCas9, the *cas9* gene is flanked by *gdh* promoter (striated box) and 3′ untranslated region of the *ran* gene (dotted box). The nuclear localization signal (filled gray box) and an HA tag (filled black box) are fused to the C terminus. In construct pNMLFtd, a single gRNA is under the control of the *Giardia* U6 promoter. pNMLFtd also has the HR template cassette composed of the *neo* selectable marker and the 5′ and 3′ flanking region of the *mlf* gene as homologous arms. The Cas9/gRNA cutting site in the genomic *mlf* gene is indicated by a red arrow. Replacement of the genomic *mlf* gene with the *neo* gene will occur by HR, after introducing a double-stranded DNA break in the *mlf* gene. The pgCas9 and pNMLFtd constructs were transfected into trophozoites. The MLFtdNeo stable transfectants were established under G418 selection. G418 was removed from the MLFtdNeo cell line to obtain the MLFtdNeo –G418 (MLF KD) cell line. The control cell line is wild-type nontransfected WB trophozoites. To complement the targeted disruption of the *mlf* gene, a pPMLF expression vector was transfected to the MLF KD cell line. pPMLF is also described in Fig. 1A. The MLF KD + MLF (Complement) cell line was established under puromycin selection to maintain the MLF expression cassette. The control cell line is MLF KD cell line transfected with 5’∆5N-Pac plasmid and selected with puromycin. **B** PCR confirmed partial replacement of the *mlf* gene with the *neo* gene in the MLF KD cell line. Genomic DNA was isolated from MLF KD and control cell lines cultured in growth medium (vegetative growth, Veg). PCR was performed using primers specific for *mlf* (PCR1 in panel A), *neo* (PCR2 in panel A), *cwp1*, *cwp2*, and *ran* genes, respectively. Products from the *cwp1*, *cwp2*, and *ran* genes are internal controls. **C** Real-time PCR confirmed partial disruption of the *mlf* gene in the MLF KD cell line. Real-time PCR was performed using primers specific for *mlf*, *cwp1*, *cwp2*, and *ran* genes, respectively. The *mlf*, *cwp1*, and *cwp2* DNA levels were normalized to the *ran* DNA level. The ratio of DNA levels in MLF KD cell line to levels in control cell line is shown and expressed as the means ± 95% confidence intervals of at least three separate experiments. **, *p* < 0.01. ns, *p* > 0.05, not significant. **D** Targeted disruption of the *mlf* gene in the MLF KD cell line resulted in decreased cyst generation during vegetative growth. Cyst number was counted from the control and MLF KD cell lines cultured in growth medium. Fold changes in cyst generation are shown as the ratio of the sum of total cysts in the MLF KD cell line relative to the control cell line. Values are shown as mean ± 95% confidence intervals. **, *p* < 0.01. **E** The CWP1 protein level decreased by targeted disruption of the *mlf* gene in the MLF KD cell line during vegetative growth. The control and MLF KD cell lines cultured in growth medium were subjected to SDS-PAGE and Western blot analysis using anti-MLF, anti-CWP1, and anti-RAN antibodies, respectively. SDS-PAGE with Coomassie Blue staining is included as a control for equal protein loading. The band intensity from triplicate Western blots was quantified using Image J as described in Fig. 1E. *, *p* < 0.05. **F** Targeted disruption of the *mlf* gene in the MLF KD cell line resulted in decreased expression of *cwp1* and *cwp2* during vegetative growth. The control and MLF KD cell lines cultured in growth medium were subjected to quantitative real-time RT-PCR analysis using primers specific for *mlf*, *cwp1*, *cwp2*, *ran*, and 18S ribosomal RNA genes, respectively, as described in Fig. 2C. ***, *p* < 0.001. ns, *p* > 0.05, not significant

Two stable transfection systems with either puromycin or neomycin selection were successfully applied for characterization of the gene function in *G. lamblia* [69, 70]. Previously we developed a CRISPR/Cas9 system with puromycin selection (Strategies 1–3)[42]. We tried to elucidate the in vivo function of the MLF protein in *G. lamblia* by a CRISPR/Cas9 with neomycin selection and a complementation system named strategy 4 (Fig. 3A). We chose the puromycin selection system as our complementation system (Fig. 3A). The combination of these two systems may provide a successful knockdown and complementation system (Fig. 3A).

**Fig. 4:**
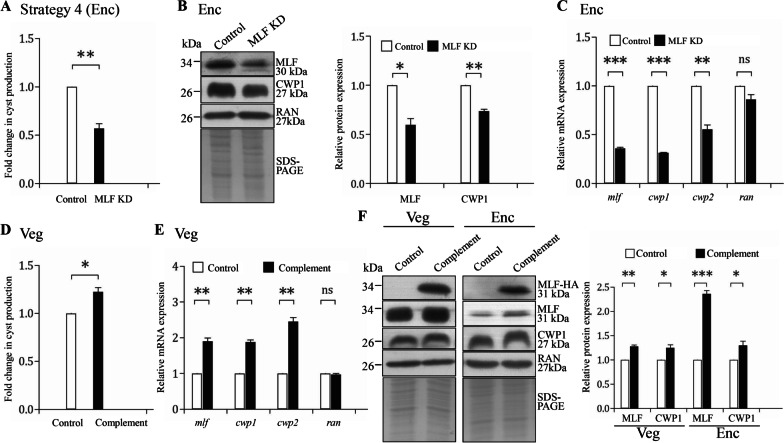
Targeted disruption and complementation of the *mlf* gene using strategy 4. **A** Targeted disruption of the *mlf* gene in the MLF KD cell line resulted in decreased cyst generation during encystation. Cyst number was counted from the control and MLF KD cell lines cultured in encystation medium for 24 h (Enc). **, *p* < 0.01. **B** The CWP1 protein level decreased by targeted disruption of the *mlf* gene in the MLF KD cell line during encystation. The control and MLF KD cell lines cultured in encystation medium for 24 h (Enc) were subjected to SDS-PAGE and Western blot analysis. The blot was probed with anti-MLF, anti-CWP1, and anti-RAN antibodies, respectively. SDS-PAGE with Coomassie Blue staining is included as a control for equal protein loading. The band intensity from triplicate Western blots was quantified using Image J as described in Fig. 1E. *, *p* < 0.05. **, *p* < 0.01. **C** Targeted disruption of the *mlf* gene in the MLF KD cell line resulted in decreased expression of *cwp1* and *cwp2* during encystation. The control and MLF KD cell lines cultured in encystation medium for 24 h (Enc) were subjected to quantitative real-time RT-PCR analysis using primers specific for *mlf*, *cwp1*, *cwp2*, *ran*, and 18S ribosomal RNA genes, respectively, as described in Fig. 2C. **, *p* < 0.01. ***, *p* < 0.001. ns, *p* > 0.05, not significant. **D** Complementation of the disrupted *mlf* gene by transfection of *mlf* expression plasmid increased cyst formation during vegetation growth. Cyst number was counted from the control and complement cell lines cultured in growth medium. *, *p* < 0.05. **E** Complementation of the disrupted *mlf* gene by transfection of *mlf* expression plasmid increased *cwp1* and *cwp2* gene expression during vegetation growth. The control and Complement cell lines cultured in growth medium were subjected to quantitative real-time RT-PCR analysis using primers specific for *mlf*, *cwp1*, *cwp2*, *ran*, and 18S ribosomal RNA genes, respectively, as described in Fig. 2C. **, *p* < 0.01. ns, *p* > 0.05, not significant. **F** Complementation of the disrupted *mlf* gene by transfection of *mlf* expression plasmid increased the CWP1 protein level during vegetative growth and encystation. The control and Complement cell lines were cultured in growth (Veg) or encystation medium for 24 h (Enc) and then subjected to SDS-PAGE and Western blot analysis. The blot was probed with anti-HA, anti-MLF, anti-CWP1, and anti-RAN antibodies, respectively. SDS-PAGE with Coomassie Blue staining is included as a control for equal protein loading. The band intensity from triplicate Western blots was quantified using Image J as described in Fig. 1E. *, *p* < 0.05. **, *p* < 0.01. ***, *p* < 0.001

We transfected *G. lamblia* trophozoites with the pgCas9 plasmid, which contains the Cas9 expression cassette, and with the pNMLFtd plasmid, which contains the gRNA expression cassette and the homologous recombination (HR) template cassette with the neomycin phosphotransferase (*neo*) selectable marker (Fig. 3A). The MLFtdNeo stable transfectants were established under G418 selection. Subsequent analysis was performed after removal of the drug for 1 month to establish the cell line with knockdown of *mlf*, which was found in our previous study (Fig. 3A)[42]. The replacement of the *mlf* gene with the *neo* gene in MLF knockdown (KD) cell line was confirmed by PCR (Fig. 3B)[42]. The results from PCR and quantitative real-time PCR show a successful disruption of the *mlf* gene by about 39% and a partial replacement of the *mlf* gene with the *neo* gene (Fig. 3C). The level of cyst formation decreased significantly in the MLF KD cell line relative to the control cell line (Fig. 3D). The levels of the MLF and CWP1 proteins significantly decreased in the MLF KD cell line relative to the control cell line during vegetative growth (Fig. 3E). We further analyzed whether the transcript levels were changed by quantitative real-time analysis, and found that the levels of *mlf*, *cwp1*, or *cwp2* mRNAs decreased significantly in the MLF KD cell line relative to the control cell line (Fig. 3F). Similar results were obtained during encystation (A, Fig. 4B, C). The results suggest a successful establishment of a CRISPR/Cas9 knockdown system with G418 selection.

### Establishment of a complementation system for the CRISPR/Cas9 system and complementation of MLF can restore *cwp1-2* gene expression and cyst formation

For establishing a complementation system, we used the pPMLF vector, which was used to overexpress MLF protein, to complement the *mlf* knockdown (Fig. 3A)[42]. We confirmed the effect of the complementation of the *mlf* gene. The level of cyst formation increased significantly in the complement cell line relative to the control cell line (Fig. 4D). We found that the levels of *mlf*, *cwp1*, or *cwp2* mRNAs, and the levels of the MLF and CWP1 proteins increased obviously in the complement cell line relative to the control cell line during vegetative growth (Fig. 4E, F). Similar results were obtained during encystation (Fig. 4F). The results suggest a successful establishment of a complementation system for CRISPR/Cas9 knockdown system.

### Nocodazole, DTT, and G418 induced ROS production and the numbers of the vesicles and levels of MLF, FYVE, and ATG8L proteins

**Fig. 5:**
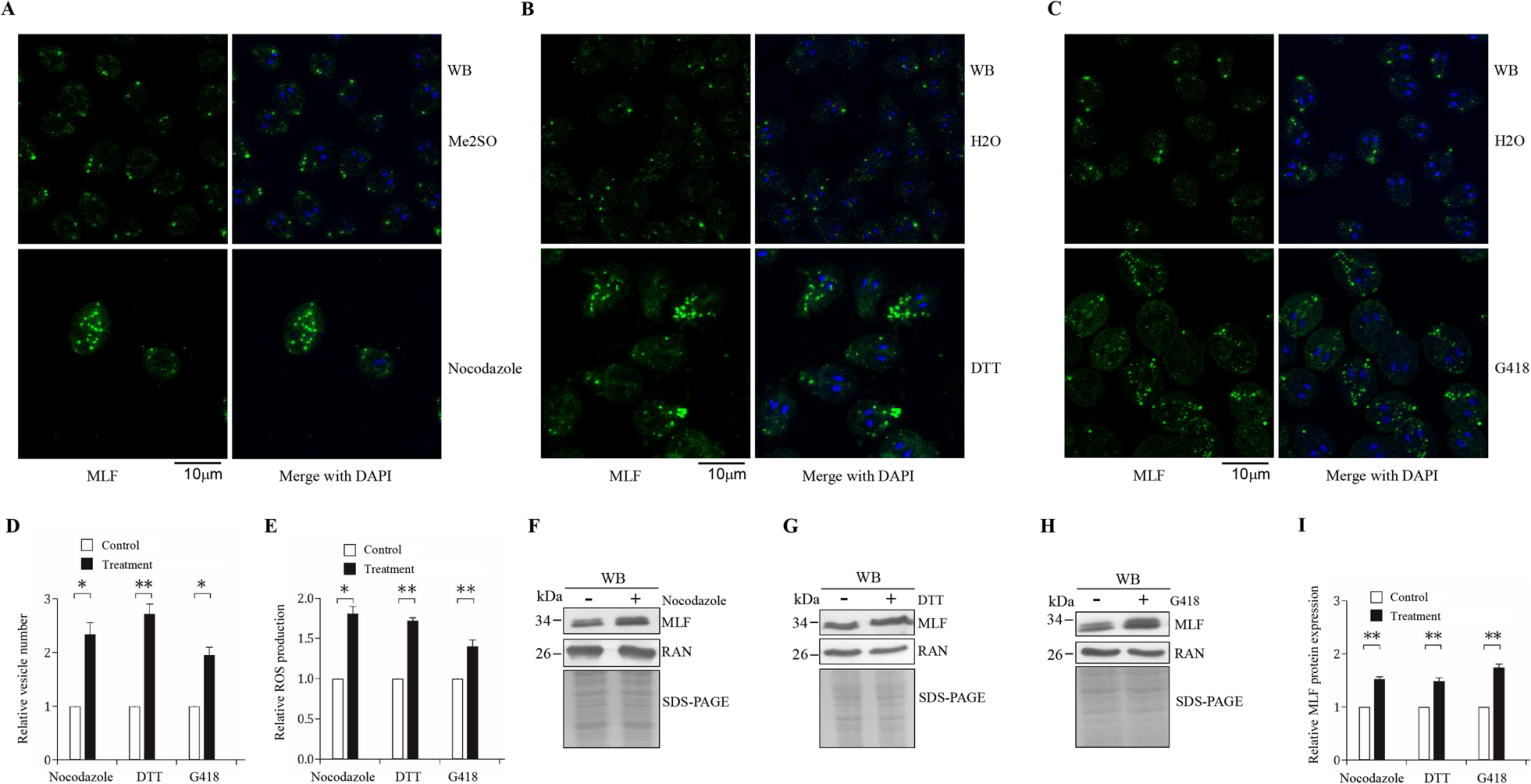
Increased numbers of MLF vesicles and levels of MLF protein and ROS production by nocodazole, DTT, and G418 treatment. **A**, **B**, **C** MLFVs can be induced by nocodazole, DTT, and G418 treatment. The wild-type non-transfected WB cells were cultured in growth medium with (A) 5 μM nocodazole, (B) 5 mM DTT, and (C) 217 μM G418, or the same volume of solvent (H2O or Me2SO) for 24 h and then subjected to immunofluorescence assay using anti-MLF antibody for detection. **D** Quantification of MLFVs in nocodazole, DTT, and G418 treated cells during vegetative stage was performed using Imaris software. *, *p* < 0.05. **, *p* < 0.01 (*n* = 200–300 cells/condition). **E** Nocodazole, DTT, and G418 treatment increased ROS production. The wild-type non-transfected WB cells were cultured in growth medium with 5 μM nocodazole, 5 mM DTT, and 217 μM G418, or the same volume of solvent (H2O or Me2SO) for 24 h and then subjected to ROS measurement. Fold change is calculated as the ratio of the difference between the treatment group and control group, to which a value of 1 was assigned. **F**, **G**, **H** Nocodazole, DTT, and G418 treatment increased the levels of MLF protein. The wild-type non-transfected WB were cultured in growth medium containing (F) 5 μM nocodazole, (G) 5 mM DTT, and (H) 217 μM G418, or the same volume of solvent (H2O or Me2SO) for 24 h and then subjected to SDS-PAGE and Western blot analysis. The blot was probed with anti-MLF and anti-RAN antibodies, respectively. SDS-PAGE with Coomassie Blue staining is included as a control for equal protein loading. **I** The band intensity from triplicate Western blots was quantified using Image J as described in Fig. 1E. **, *p* < 0.01

It has been known that ROS may activate autophagy for inhibition of ROS-induced damage in leukemia cells [29]. If MLF plays a role in aberrant protein degradation or autophagy, it might be upregulated in response to various stress inducers that cause ROS production and commonly affect autophagy. It has been revealed that the MLF protein was detected in cytosolic vesicles (MLFVs) whose numbers increased by encystation or MG132-, rapamycin-, and chloroquine- mediated stress [47]. We tried to understand whether MLF was stress-induced using three other stress-related drugs, nocodazole, DTT, and G418, which are well-known ROS inducers and contributes to increased autophagosomes in mammalian cells [56, 62–64, 71, 72]. We found that Nocodazole, DTT, and G418 treatment increased the number of MLFVs (Fig. 5A–D). The same treatment also significantly increased the ROS production and MLF protein levels (Fig. 5E–I). ROS was also induced by MG132, rapamycin, and chloroquine, which increased MLF protein and vesicles (Additional file 1: Fig. S1)[47]. The induction of MLF protein levels by the above agents suggests a positive role for MLF in response to stress and ROS production.

**Fig. 6:**
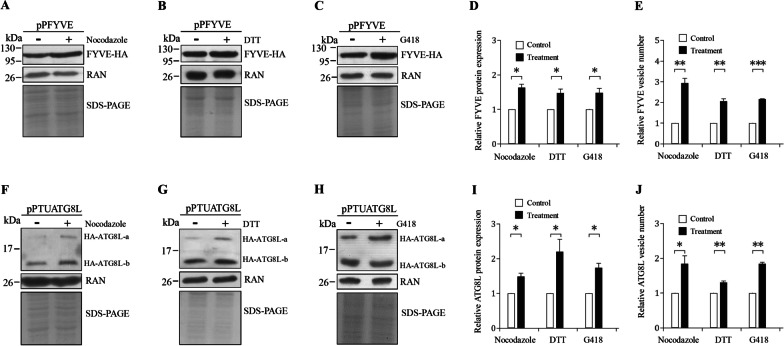
Increased levels of FYVE and ATG8L proteins and numbers of their vesicles by nocodazole, DTT, and G418 treatment. **A**, **B**, **C** Nocodazole, DTT, and G418 treatment increased the level of FYVE protein. The pPFYVE stable transfectants were cultured in growth medium containing (A) 5 μM nocodazole, (B) 5 mM DTT, and (C) 217 μM G418, or the same volume of solvent (H2O or Me2SO) for 24 h and then subjected to SDS-PAGE and Western blot analysis. The blot was probed with anti-HA and anti-RAN antibodies, respectively. SDS-PAGE with Coomassie Blue staining is included as a control for equal protein loading. **D** The band intensity from triplicate Western blots of FYVE experiments was quantified using Image J as described in Fig. 1E. *, *p* < 0.05. **E** FYVE-localized vesicles can be induced by nocodazole, DTT, and G418 treatment. The pPFYVE stable transfectants were cultured in growth medium with 5 μM nocodazole, 5 mM DTT, and 217 μM G418, or the same volume of solvent (H2O or Me2SO) for 24 h and then subjected to immunofluorescence assay using anti-HA antibody for detection. Quantification of FYVE-localized vesicles in nocodazole, DTT, and G418 treated cells during vegetative stage was performed using Imaris software. **, *p* < 0.01 (*n* = 200–300 cells/condition). **F**, **G**, **H** Nocodazole, DTT, and G418 treatment increased the level of ATG8L protein. The pPTUATG8L stable transfectants were treated with (F) nocodazole, (G) DTT, and (H) G418 and then subjected to SDS-PAGE and Western blot analysis. The blot was probed with anti-HA and anti-RAN antibodies, respectively. SDS-PAGE with Coomassie Blue staining is included as a control for equal protein loading. **I** The band intensity from triplicate Western blots of ATG8L experiments was quantified using Image J as described in Fig. 1E. *, *p* < 0.05. **J** ATG8L-localized vesicles can be induced by nocodazole, DTT, and G418 treatment. The pPTUATG8L stable transfectants were treated with nocodazole, DTT, and G418 and then subjected to immunofluorescence assay using anti-HA antibody for detection. Quantification of ATG8L-localized vesicles in nocodazole, DTT, and G418 treated cells during vegetative stage was performed using Imaris software. *, *p* < 0.05. **, *p* < 0.01 (*n* = 200–300 cells/condition)

Similarly, Nocodazole, DTT, and G418 treatment also increased the levels of FYVE and ATG8L proteins and the numbers of FYVE- or ATG8L-localized vesicles (Fig. 6A–J, Additional file 1: Fig. S2), suggesting a positive role of FYVE and ATG8L in the response to stresses.

### CDK2m3 was increased by stresses and decreased with downregulated MLF

**Fig. 7:**
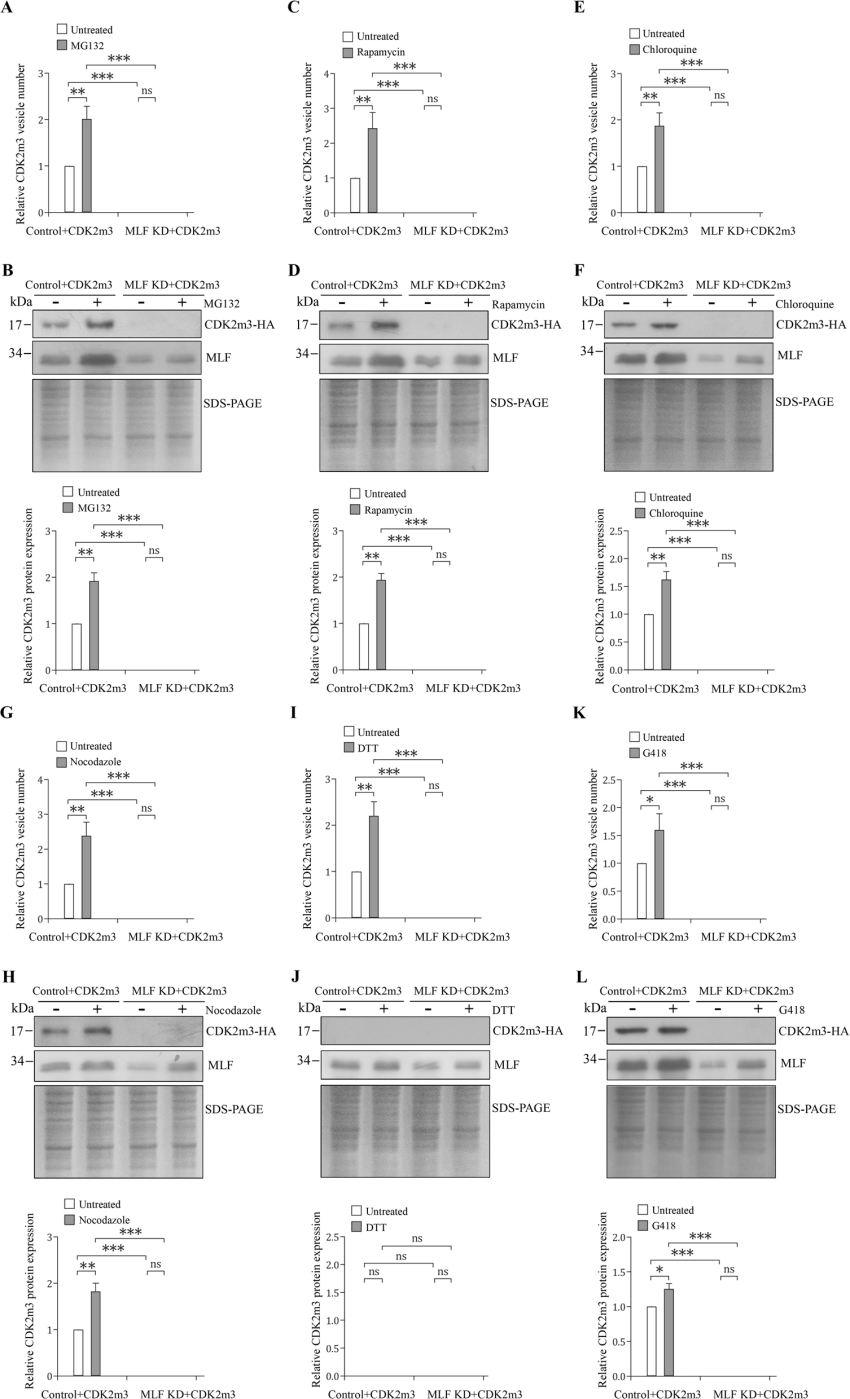
The levels of CDK2m3 mutant protein and vesicles decreased in the MLF knockdown trophozoites. **A**, **C**, **E**, **G**, I, **K** Quantification of Cdk2m3 vesicles in the MLF knockdown trophozoites. A pPCdk2m3 expression vector was transfected to the MLF KD and control cell lines (trophozoites stably transfected with pRANneo vector with further removal of G418). The stable transfectants were cultured in growth medium containing (A) 80 μM MG132, (C) 36 μM rapamycin, (E) 100 μM chloroquine, (G) 5 μM nocodazole, (I) 5 mM DTT, and (K) 217 μM G418 for 24 h and then subjected to immunofluorescence analysis using anti-HA antibody for detection. Quantification of CDK2m3-localized vesicles in the treated cells during vegetative stage was performed using Imaris software. **, *p* < 0.01. ***, *p* < 0.001 (*n* = 200–300 cells/condition). ns, *p* > 0.05, not significant. **B**, **D**, **F**, **H**, **J**, **L** The level of Cdk2m3 protein decreased in the MLF knockdown trophozoites. The MLF KD and control cell lines were used to transfect pPCdk2m3. The stable transfectants were subcultured in growth medium containing (B) 80 μM MG132, (D) 36 μM rapamycin, (F) 100 μM chloroquine, (H) 5 μM nocodazole, (J) 5 mM DTT, and (L) 217 μM G418 for 24 h and then subjected to SDS-PAGE and Western blot analysis. The blot was probed with anti-HA and anti-MLF antibodies, respectively. SDS-PAGE with Coomassie Blue staining is included as a control for equal protein loading. The band intensity from triplicate Western blots was quantified using Image J. The CDK2m3 protein levels were normalized to the loading control (Coomassie Blue-stained proteins). The ratio of CDK2m3 protein levels in drug-treated sample to levels in untreated sample is shown and expressed as mean ± 95% confidence intervals. *, *p* < 0.05. **, *p* < 0.01. ***, *p* < 0.001. ns, *p* > 0.05, not significant

A signal transducer, CDK2, can induce cyst formation and localize to cytoplasm in *Giardia* [46]. Deletion of a part of kinase domain caused loss of ability for cyst induction and mislocalization of CDK2m3 to cytosolic vesicles that colocalized with MLF [47]. Previously we used this CDK2 mutant protein as a model to study protein metabolism [47]. Inhibition of either proteasomes or autophagy interferes with clearance of aberrant proteins in mammalian and tumor cells [73, 74]. The increase of ubiquitination of CDK2m3 by MG132 treatment suggests it is degraded by proteasome [47]. Further findings, including the increased CDK2m3 vesicles and MLFVs by MG132, rapamycin, and chloroquine treatment, the colocalization of CDK2m3 with MLFVs, and the interaction of CDK2m3 and MLF, suggest that MLF is involved in CDK2m3 metabolism from the proteasome or autophagy pathway [47]. It has been known that autophagy plays a normal role in removing misfolded or aggregated proteins and that its dysfunction may result in stress conditions, including oxidative stress [75, 76]. We tested several agents that generate oxidative stress and increase autophagosomes in mammalian cells, yeast, and plants, including MG132, rapamycin, chloroquine, nocodazole, DTT, and G418, in *G. lamblia* [53–64]. Treatment with five stress inducers increased the CDK2m3 protein levels and vesicles in the control cells (Fig. 7A–L, Additional file 1: Fig. S3). Treatment with DTT also increased the number of CDK2m3 vesicles in the control cells (Fig. 7I, Additional file 1: Fig. S3). The effect of DTT on CDK2m3 proteins is not sure, because no detection of CDK2m3 protein in untreated or DTT treated sample (Fig. 7J). Knockdown of MLF significantly reduced the CDK2m3 protein and vesicles to undetectable levels even after treatment with the five stress inducers (Fig. 7A–L). In addition, we found that transfection of MLF expression vector increased CDK2m3 protein level, suggesting that MLF can positively regulate CDK2m3 (Additional file 1: Fig. S4). Collectively, our findings suggest that the mutant protein, CDK2m3, is significantly increased in a stress-induced cellular model. The findings also suggest that MLF plays a positive role in stress-induced CDK2m3 formation.

### Treatment with 3-MA decreased CDK2m3 and MLF proteins and vesicles

**Fig. 8:**
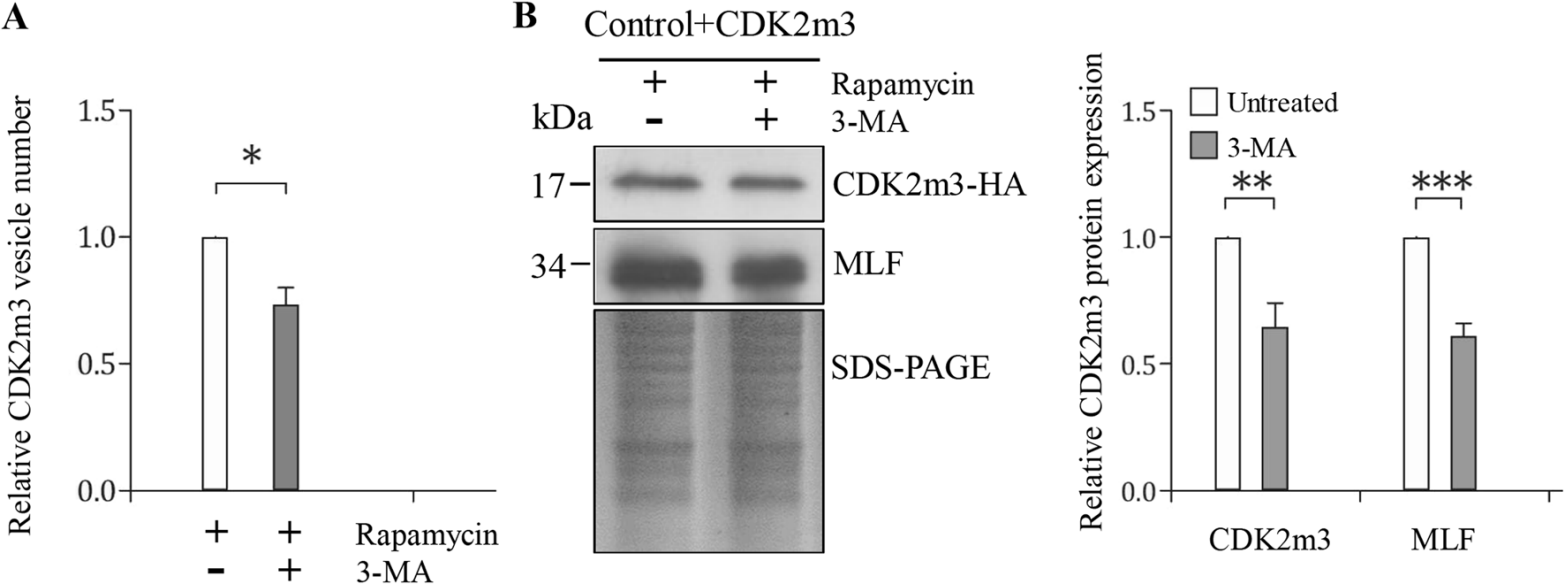
The levels of CDK2m3 mutant protein and vesicles decreased in response to 3-MA treatment. **A** Quantification of Cdk2m3 vesicles in the 3-MA treatment. The pPCdk2m3 stable transfectants were treated with 36 μM rapamycin in the absence or presence of 5.6 mM 3-MA for 24 h and then subjected to immunofluorescence analysis using anti-HA antibody for detection. Quantitation of vesicles was performed using Imaris software. *, *p* < 0.05. (*n* = 200–300 cells/condition). **B** The level of Cdk2m3 protein decreased in response to 3-MA treatment. The pPCdk2m3 stable transfectants were treated with 36 μM rapamycin in the absence or presence of 5.6 mM 3-MA for 24 h and then subjected to SDS-PAGE and Western blot analysis. The blot was probed with anti-HA, anti-MLF, and anti-RAN antibodies, respectively. SDS-PAGE with Coomassie Blue staining is included as a control for equal protein loading. The band intensity from triplicate Western blots was quantified using Image J. The CDK2m3 and MLF protein levels were normalized to the loading control (Coomassie Blue-stained proteins). The ratio of CDK2m3/MLF protein levels in 3-MA-treated sample to levels in untreated sample is shown and expressed as mean ± 95% confidence intervals. **, *p* < 0.01. ***, *p* < 0.001

3-MA is a phosphatidylinositol 3-kinase (PI3K) inhibitor, which has been often used as an autophagy inhibitor as it can prevent the activation of autophagy and decrease autophagosome number in mammalian cells [77, 78]. To understand whether CDK2m3 metabolism are related to autophagy, we tested the effect of 3-MA on the CDK2m3 accumulation. The CDK2m3 cell line was treated with rapamycin in the absence or presence of 3-MA. Interestingly, the addition of 3-MA significantly suppressed the rapamycin-induced CDK2m3 and MLF proteins and vesicles (Fig. 8A, B). This suggests that 3-MA has negative effects on CDK2m3 and MLF, and that these proteins could be in the autophagy-related clearance pathway.

### Increased drug sensitivity of the *mlf* targeted disruption cell line and complementation of the disrupted *mlf* gene recovered cell growth after stress

**Fig. 9:**
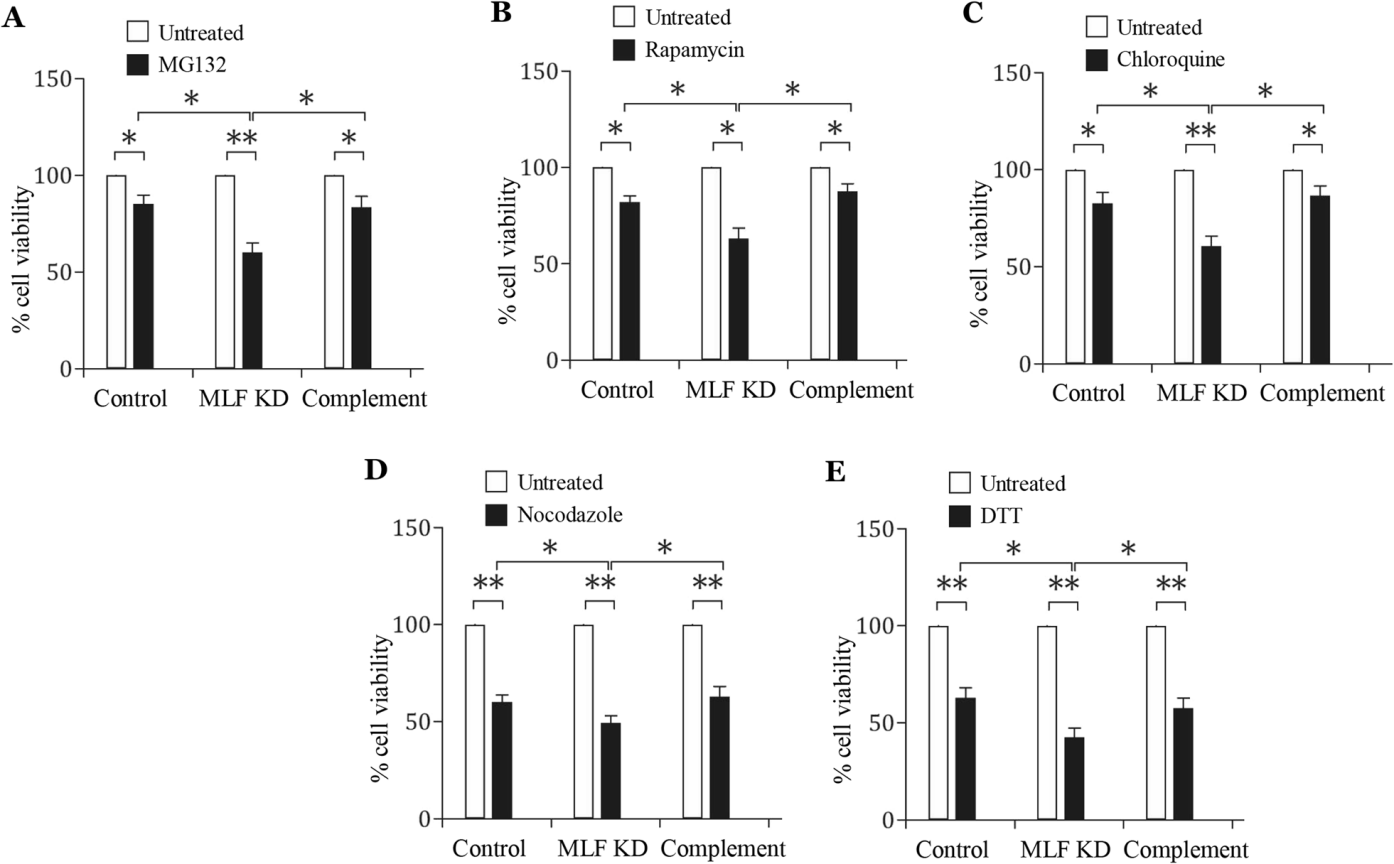
Complementation of the disrupted *mlf* gene by transfection of MLF expression plasmid recovered cell growth after stress. **A**, **B**, **C**, **D**, **E** To complement the disrupted *mlf* gene, a pPMLF expression vector was transfected to the MLF KD and control cell lines. The complement and control cell lines were subcultured in growth medium containing (A) 80 μM MG132, (B) 36 μM rapamycin, (C) 100 μM chloroquine, (D) 5 μM nocodazole, and (E) 5 mM DTT for 24 h and then subjected to cell count. An equal volume of solvent (H2O or Me2SO) was added to cultures as an untreated control. Fold changes in cell number are shown as the ratio of cell number in the treatment relative to the control group. Values are shown as mean ± 95% confidence intervals of three independent experiments. *, *p* < 0.05. **, *p* < 0.01

To investigate whether MLF is related to tolerance to stresses in *G. lamblia*, we also used the above stress inducers to test the drug sensitivity of the MLF KD cell line. All six stress inducers had growth-inhibiting effect on the control cells (Fig. 9A–E). Interestingly, the viability of the MLF KD cell line decreased compared to the control cell line after MG132, rapamycin, chloroquine, nocodazole, and DTT treatment, suggesting that MLF KD cell line exhibited increased sensitivity to these stress inducers (Fig. 9A–E). G418 was used to select and generate the stable cell lines for MLF KD (Fig. 3A), and was thereby not shown as it did not affect the growth of MLF KD cell line. Complementation of MLF KD cells with MLF expression plasmid reduced sensitivity to the stress inducers (Fig. 9A–E), suggesting that expression of MLF may decrease toxic effect. Our findings suggest that MLF is important for survival in various stress conditions.

## Discussion

**Fig. 10:**
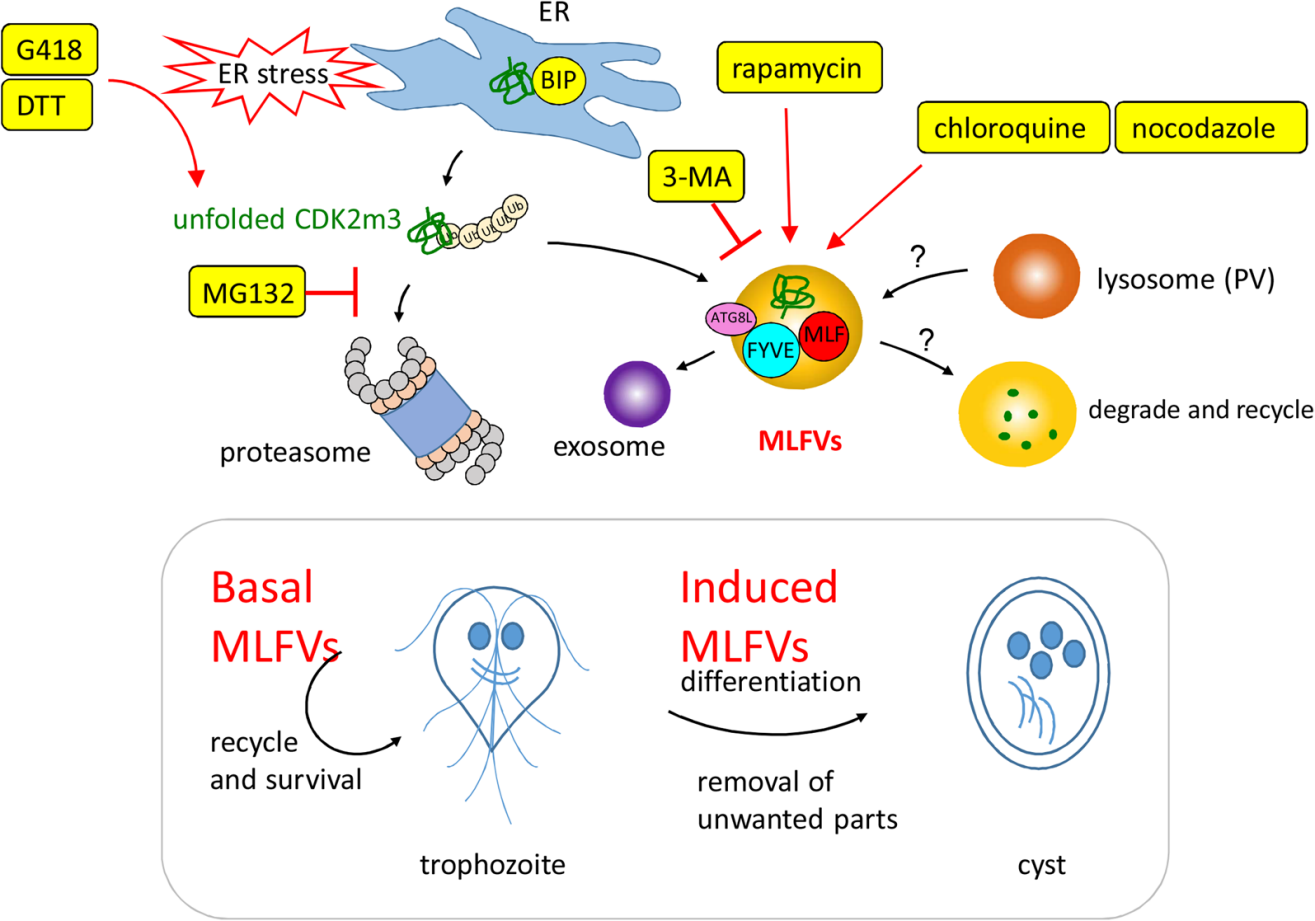
MLFV compartments are induced by six autophagosome-inducing agents, but decreased by 3-MA, an autophagosome-reducing agent. MLF interacts with FVYE and ATG8L in their compartments. MLFVs are maintained at a basal level in vegetative trophozoites but increases during encystation. During encystation, unwanted trophozoite specific proteins are accumulated. Accumulation of toxic undegraded proteins may induce protein clearance pathway to promote cell survival during encystation. Therefore, MLF, FVYE, and ATG8L, are induced for cooperation in protein clearance during encystation. BIP, as a chaperone, binds to unfolded proteins and helps refold the proteins to a soluble form. Unfolded proteins can also be degraded by ubiquitin–proteasome systems. In the presence of stress inducers, excess of misfolded proteins may overwhelm the capacity of chaperone and proteasome systems and lead to toxicity to cells. The aberrant protein, CDK2m3, may be degraded by specific clearance pathway through entering MLFV compartments. The compartments may fuse with lysosomes (peripheral vesicles, PV) to recycle materials and control energy balance. Treatment with chloroquine or nocodazole, an inhibitor of fusion of the compartments and lysosome, may impair the process and lead to accumulation of aberrant proteins, such as CDK2m3. Treatment with MG132, a proteasome inhibitor, may impair protein quality control, leading to unfolded protein response (UPR). Treatment with rapamycin, an autophagy inducer, may activate UPR. Treatment with DTT, an ER stress inducer, may activate UPR. Treatment with G418, an aminoglycoside antibiotic that interferes folding of proteins, may generate UPR. UPR causes accumulation of aberrant proteins, such as CDK2m3. All the six agents that promote autophagosome accumulation in higher eukaryotes, can induce MLF protein and vesicles in *G. lamblia*. Five can induce CDK2m3 protein and vesicles. 3-MA, an autophagosome-reducing agent in higher eukaryotes, can reduce CDK2m3 and MLF proteins and vesicles in *G. lamblia*. Our findings suggest that MLFVs involve in the clearance of CDK2m3 during stresses and share similar characteristics with autophagosomes

Autophagy is used by eukaryotic cells to cope with the stress caused by the toxic agents [27]. Toxic insults, such as ER stress and ROS, result in accumulation of aberrant-folded protein and protective induction of autophagy [27, 64]. Previously we found that MLF is a stress response protein that is induced during proteasomal inhibition and autophagy induction and localizes to cytoplasmic vesicles (MLFVs)[47]. CDK2m3 was used as a cargo marker to identify the mechanism of misfolded protein [47]. MG132 treatment increased ubiquitination of the CDK2m3 protein [47]. In addition, CDK2m3 can induce MLF expression, colocalize and interact with MLF in cytoplasmic vesicles (MLFVs)[47]. It is of interest to understand the role of MLF in the metabolism of CDK2m3. Here we demonstrate that the levels of CDK2m3 protein in the MLF KD cells are undetectable even after stress induction (Fig. 7), indicating that MLF promotes CDK2m3 formation. We found that the MLF levels could reflect the protein metabolism status since CDK2m3 formation was decreased in MLF knockdown cells and increased when MLF was overexpressed (Fig. 7, Additional file 1: Fig. S4). We hypothesize that MLF could bind to the aberrant proteins, mark them for degradation, and recruit components of the degradative machinery. MLFVs could act as stress-induced compartments for substrates of the proteasome and autophagy, like conventional autophagosomes bearing protein cargos (Fig. 10). Similarly, it has been shown that human MLF2 may function in maintaining protein stability and vesicle trafficking [41, 65, 79].

Proteins utilize the proteasome pathway for degradation may be increased by the treatment with proteasome inhibitor [73]. It has been shown that treatment with proteasome inhibitors not only increases accumulation of aberrant protein, but also induces autophagy and increases the number of autophagosomes [59, 80]. We found that treatment with the proteasome inhibitor MG132 can increase CDK2m3 levels and vesicles, indicating that CDK2m3 is degraded by proteasome (Fig. 7)[47]. Addition of MG132 also increases the MLF protein level, as well as the number of MLFVs [47]. However, the levels of CDK2m3 protein in the MLF KD cells were undetectable even after MG132 treatment (Fig. 7). The results suggest that MLF is involved in MG132-induced CDK2m3 accumulation. One possible explanation is that MLF protects CDK2m3 from degradation by proteasome. Another explanation is that MLF could enhance CDK2m3 accumulation in response to MG132-induced stress (see below).

MLF and CDK2m3 were co-localized in MLFVs in *G. lamblia* [47]. To establish whether CDK2m3 is, in fact, differentially targeted to degradative compartments, MLFVs, we treated cells with the lysosomal inhibitor chloroquine, a weak base that increased the pH of acidic lysosomes in mammals [60, 81]. Degradation of autophagic proteins will be inhibited by chloroquine which can block the fusion of autophagosomes with lysosomes and thereby increase the number of the autophagosomes in mammalian and tumor cells [60, 82–84]. As predicted, CDK2m3 protein and vesicles increased by addition of chloroquine in *G. lamblia* (Fig. 7)[47], indicating that CDK2m3 is degraded by lysosome. Addition of chloroquine also increases the MLF protein level, as well as the number of MLFVs. However, the CDK2m3 protein was decreased to an undetectable level by MLF KD, even after chloroquine treatment (Fig. 7). The results suggest that MLF is involved in chloroquine-induced CDK2m3 accumulation. One possible explanation is that MLF protects CDK2m3 from degradation by lysosome. Another explanation is that MLF could enhance CDK2m3 accumulation in response to chloroquine-induced stress (see below).

Nocodazole is another agent that has been reported to inhibit autophagosome-lysosome fusion in mammalian cells [61]. It can inhibit microtubule polymerization, increase the autophagosome number, and inhibit autophagy-mediated protein degradation [61]. Similar to the result from chloroquine treatment, the CDK2m3 protein and vesicles increased by addition of nocodazole and decreased by MLF KD in *G. lamblia* (Fig. 7), suggesting that microtubule dynamics are important for CDK2m3 metabolism.

The above autophagosome-inducing agents, MG132, chloroquine, and nocodazole, can increase the amount of MLF and CDK2m3 proteins and vesicles in *G. lamblia* (Fig. 7) [47, 59–61]. Rapamycin is an autophagy inducer and it can increase the number of autophagosomes in mammalian cells [62]. DTT can induce ER stress that triggers autophagy in mammalian cells, yeast, and plants [64, 84, 85]. G418 belongs to aminoglycoside antibiotics that inhibit eukaryotic and prokaryotic protein synthesis and interfere correct translation and posttranslational folding of proteins [56, 86]. Therefore, aminoglycosides are inducers of autophagy in mammalian cells [63]. We found that the mammalian autophagosome-inducing agents, including rapamycin, DTT, and G418 [62–64], increased the amount of MLF protein and vesicles in *G. lamblia* (Figs. 5, 7). Rapamycin and G418 also increased the amount of CDK2m3 protein and vesicles (Fig. 7). Although DTT can increase MLF protein (Fig. 5), it is still unsure whether DTT affects the CDK2m3 protein (Fig. 7). The no detection of CDK2m3 protein in both the untreated or DTT treated sample suggests that the untreated sample was affected by DTT that diffused from the next lane [87]. It has been suggested that DTT may generate protein degradation in the presence of ROS in purified protein samples [88].

Various agents can cause oxidative stress, a phenomenon that ROS may overwhelm the capacity of antioxidant defense systems in mammals and yeast [27, 89]. Various stress conditions, such as oxidative stress and ER stress, may promote accumulation of ubiquitinated aberrant proteins in mammalian cells and yeast [90, 91]. Therefore, we used the mutant protein, CDK2m3, as a model for testing the above stress response. DTT can induce ER stress that activates unfolded protein response (UPR) in response to an accumulation of unfolded proteins in plants [85]. DTT-induced UPR may induce activation of autophagy and ROS production in mammals, yeast, and plants [53, 64, 84, 85]. MG132 inhibits proteasome and also increases ER stress, oxidative stress, and ROS production in mammalian and tumor cells [54, 92, 93]. Rapamycin induces ROS levels, UPR, and autophagy in mammalian and tumor cells [55, 62, 94, 95]. G418 is an aminoglycoside antibiotic that may inhibit protein synthesis and interfere folding of proteins in mammalian cells [56, 86], and generate ROS production, oxidative stress, ER stress, and UPR in mammals [56, 96]. Nocodazole induces UPR and ROS generation in mammalian cells and yeast [57, 71, 97]. Chloroquine induces oxidative stress and increases the intracellular level of ROS in mammalian cells [58, 98]. All the six drugs induced ROS production (Fig. 5, Additional file 1: Fig. S1), suggesting that they can induce oxidative stress in *G. lamblia*. MLF KD decreased the amount of CDK2m3 protein and vesicles in *G. lamblia* treated with five drugs (Fig. 7), suggesting that MLF could enhance CDK2m3 accumulation in response to oxidative stress, ER stress, or UPR.

Previously we found that distinct autophagy inducers, such as starvation and rapamycin, stimulated the MLF expression [47]. In addition, MLF colocalized with the autophagy-related proteins, FYVE and ATG8L [47]. Our tested autophagosomes-inducing drugs, including MG132, rapamycin, chloroquine, nocodazole, DTT, and G418, not only induced the upregulation of MLF, they also induced its binding partners, FYVE and ATG8L (Fig. 6)[47], suggesting common and specific roles of MLF, FYVE, and ATG8L in the autophagy-related response. It is possible that these three proteins and their compartments are upregulated for degradation of accumulated unfolded proteins by the addition of the autophagosomes-inducing drugs (Fig. 10). Previously we also found that these three proteins and their compartments are upregulated in encystation stage (Fig. 10)[47], suggesting that the compartments are needed to degrade unwanted trophozoite-specific proteins during encystation. Likewise, encystation (cryptobiosis) and concurrent formation of autophagosomes in cilliates can be induced by nutrient starvation, a typical inducer of autophagy [34]. On the other hand, the addition of PI3K inhibitor/autophagy inhibitor/autophagosome reducing agent, 3-MA, suppressed the rapamycin-induced MLF and CDK2m3 proteins and vesicles, which is correlated with the negative effect of 3-MA on the autophagic responses in other eukaryotes [77, 78]. The finding suggests that the MLF-related protein metabolism is activated by a PI3K-dependent mechanism. In conclusion, autophagosome-inducing and reducing agents have positive and negative effect on the MLF and CDK2m3 proteins and vesicles, respectively. This suggests that the MLFVs/CDK2m3 vesicles and autophagosomes may have similar fates or functions.

*Giardia* MLF is the only MLF family member found in the protozoan parasite [43]. Previously we demonstrated that *Giardia* MLF has moderate similarity to the human MLF1 and MLF2 [42]. Like human and *Drosophila* MLF that play critical roles in blood cell differentiation, we also found that *Giardia* MLF induces cyst differentiation [15, 42, 43]. To understand MLF function change in evolution, we tested whether human MLF2 had similar inducing effect with *Giardia* MLF on cyst differentiation. Interestingly, we found that hMLF2 colocalized with *Giardia* MLF in vesicles, interacted with *Giardia* MLF, and had significant encystation-inducing activity (Fig. 1), suggesting that MLF protein family is functionally conserved. The presence of MLF family protein in *G. lamblia* suggests that MLF protein appeared early in eukaryotic evolution. Human MLFs has ability to maintain protein stability and regulate unfolded and aggregated protein [41, 44, 99]. *Giardia* MLF may play a role in protein metabolism since MLF levels reflect the levels of CDK2 mutant protein. Similarly, the steady state levels of the scaffold protein p62 could reflect the autophagic status since it recruits protein substrates to autophagosomes by interacting with light chain 3 [100]. Another example is chaperone, whose levels reflect the status of protein folding in cells [101].

CRISPR/Cas9 system has been shown as a useful method for target disruption in *G. lamblia* [19, 42, 102, 103]. For our gene disruption strategy, a plasmid consists of a selection marker cassette flanking with two homologous sequences, is transfected into *G. lamblia* with a transient plasmid consisting of Cas9 expression cassette (Fig. 3)[42]. The target gene can be replaced by the selection marker gene through HR (Fig. 3)[42]. Another selection marker gene can be used to express the gene for complementation. In our case, we used the neomycin and puromycin resistance genes for CRISPR/Cas9 and complementation, respectively (Fig. 3). This system may be helpful for investigating the gene function in *G. lamblia*.

## Conclusions

Our studies provide evidence that MLF protein family is functionally conserved in eukaryotic evolution. MLF could render tolerance to various stresses and involve in stress-induced CDK2m3 accumulation. MLF-related protein metabolism is similar to autophagy, affects cell differentiation and stress tolerance in *G. lamblia*, suggesting that the related studies provides valuable insights for of drug development. The combination of the two drug selection systems also provide a successful knockdown and complementation system, which is an emerging tool for examining the biological role of a target gene in *G. lamblia*.

## Methods

### *G. lamblia* culture

Trophozoites of *G. lamblia* WB, clone C6 (see ATCC 50,803)(obtained from ATCC), were cultured in modified TYI-S33 medium [104]. Encystation was performed as previously described [12]. Briefly, trophozoites grown to late log phase in growth medium were harvested and encysted for 24 h in TYI-S-33 medium containing 12.5 mg/ml bovine bile at pH 7.8 at a beginning density of 5 × 10^5^ cells/ml. In experiments exposing *G. lamblia* vegetative trophozoites to different drugs, WB clone C6 trophozoites were cultured in growth medium with 80μ M MG132, 36 μM rapamycin, 100 μM chloroquine, 5 μM nocodazole, 50 mM DTT, or 217 μM G418 for 24 h.

*Cyst count* Cyst count was performed as previously described [43, 47].

### RNA extraction, RT-PCR and quantitative real-time PCR analysis

Synthetic oligonucleotides used are shown in Additional file 1: Table S5. Total RNA was extracted from *G. lamblia* cell line during vegetative or encystation stages using TRIzol reagent (Invitrogen). For RT-PCR, 5 μg of DNase-treated total RNA was mixed with oligo (dT)12–18 and random hexamers and Superscript II RNase H^−^ reverse transcriptase (Invitrogen). Synthesized cDNA was used as a template in subsequent PCR. For quantitative real-time PCR, SYBR Green PCR master mixture was used (Kapa Biosystems). PCR was performed using an Applied Biosystems PRISMTM 7900 Sequence Detection System (Applied Biosystems). Specific primers were designed for detection of the *mlf* (**XM_001706985.1**, open reading frame 16,424), *cwp1* (**U09330**, open reading frame 5638), *cwp2* (**U28965**, open reading frame 5435), *ran* (**U02589**, open reading frame 15,869), and 18S ribosomal RNA (**M54878**, open reading frame r0019) genes: mlfrealF and mlfrealR; cwp1realF and cwp1realR; cwp2realF and cwp2realR; ranrealF and ranrealR; 18SrealF and 18SrealR. Each primer pairs were determined for amplification efficiency ~ 95% based on the slope of the standard curve. Two independently generated stably transfected lines were made from each construct and each of these cell lines was assayed three separate times. The results are expressed as a relative expression level over control. Transcript levels were normalized to 18S ribosomal RNA levels. Results are expressed as the means ± 95% confidence intervals of at least three separate experiments. Student’s *t*-tests were used to determine statistical significance of differences between samples.

### Plasmid construction

All constructs were verified by DNA sequencing with a BigDye Terminator 3.1 DNA Sequencing kit and an Applied Biosystems 3100 DNA Analyzer (Applied Biosystems). Plasmid 5’Δ5N-Pac was a gift from Dr. Steven Singer and Dr. Theodore Nash [69]. Plasmids pPMLF, pPCDK2m3, pRANneo, pMLFko, and pgCas9 has been described previously [42, 47, 70]. To make construct pPMLFm, the *mlf* gene was amplified using two primer pairs, mlfmF and MLFMR, and mlfmR and MLFNF. The two PCR products were purified and used as templates for a second PCR. The second PCR also included primers MLFNF and MLFMR, and the product was digested with NheI and MluI and cloned into the NheI and MluI digested pPop2NHA [105]. To make construct pPhMLF2, we used gene synthesis services from IDT to obtain the hMLF2 fragment that consists of *Giardia mlf* 5' untranslated region and *hmlf2* gene flanked with NheI and MluI sites (Additional file 1: Table S3). The hMLF2 fragment was digested with NheI/MluI and cloned into NheI/MluI digested pPop2NHA [105]. The resulting plasmid, pPhMLF2, contained the *hmlf2* gene controlled by *Giardia mlf* promoter with an HA tag fused at its C-terminus. To make construct pNMLFtd, the *neo* gene was amplified from the pRANneo plasmid using PCR with primers neomF and neoXR and primers neomR and neoNF. A nucleotide in the *neo* gene is mutated to remove the NcoI site without influencing the amino acid sequence. A second run of PCR with the above two products and primers neoNF and neoXR generated a 0.8-kb PCR product that was digested with NcoI and XhoI, and cloned into NcoI/XhoI-digested pMLFko. The resulting plasmid, pNMLFtd, contains a *neo* gene as a selection marker and gRNA. The single gRNA, which is located upstream three nucleotides of protospacer-adjacent motif (NGG sequence), includes a guide sequence targeting 20-nucleotide of the *mlf* gene (nt 61–80)[42].

### Expression and purification of recombinant hMLF2 protein

The hMLF2 gene was amplified from hMLF2 synthetic fragment using oligonucleotides hMLF2F and hMLF2R. The product was cloned into the expression vector pET101/D-TOPO (Invitrogen) in frame with the C-terminal His and V5 tags to generate plasmid phMLF2. The phMLF2 plasmid was transformed into *Escherichia coli* and purified as previously described [16]. Protein purity and concentration were estimated by Coomassie Blue and silver staining compared with serum albumin. hMLF2 was purified to apparent homogeneity (> 95%).

### Transfection and western blot analysis

For CRISPR/Cas9 system in strategy 4, *G. lamblia* trophozoites were transfected with plasmid pNMLFtd and pgCas9, and then selected in 217 μM G418. The culture medium in the first replenishment contained 6 μM Scr7 and the same concentrations of G418 [42]. SCR7 is an NHEJ inhibitor for increasing HR [42]. The MLFtdNeo stable transfectants were established after selection with G418. Stable transfectants were maintained at the same concentrations of antibiotics. G418 was then removed from the medium for each stable cell line to obtain MLFtdNeo –G418 (MLF KD) cell line. The control cell line is wild-type *G. lamblia* WB trophozoites. Subsequent analysis was performed after the removal of the drug for 1 month. Stable transfectants were further analyzed by Western blotting, or DNA/RNA extraction as previously described [42]. The replacement of the *mlf* gene with the *neo* gene was confirmed by PCR and sequencing as previously described [42]. Western blots were probed with anti-V5-HRP (Invitrogen), anti-HA monoclonal antibody (1/5000 in blocking buffer; Sigma), anti-MLF (1/10,000 in blocking buffer)[42], anti-CWP1 (1/10,000 in blocking buffer)[15], anti-RAN (1/10,000 in blocking buffer)[106], or preimmune serum (1/5000 in blocking buffer), and detected with HRP-conjugated goat anti-mouse IgG (1/5000; Pierce) or HRP-conjugated goat anti-rabbit IgG (1/5000; Pierce) and enhanced chemiluminescence (Merck Millipore). The intensity of bands from three Western blot assays was quantified using Image J. The ratio of specific proteins over the loading control (RAN or Coomassie Blue-stained proteins) is calculated. Fold change is calculated as the ratio of the difference between the specific cell line and control cell line, to which a value of 1 was assigned. Results are expressed as means ± 95% confidence intervals.

### Immunofluorescence assay

The stable cell lines were cultured in growth medium under puromycin selection. Cells cultured in growth medium with or without indicated drugs, or encystation medium for 24 h were harvested and subjected to immunofluorescence assay as previously described [47, 107]. Cells were reacted with anti-MLF (1/300 in blocking buffer)[42] or Anti-HA monoclonal antibody (1/300 in blocking buffer; Covance). Anti-rabbit ALEXA 568 or anti-mouse ALEXA 488 (1/500 in blocking buffer, Life Technologies) was used as the detector. The protein localization was visualized using a Leica TCS SP5 spectral confocal system. Images were analyzed by Imaris software (Bitplane). Student’s t test was used to perform statistical analysis (*, *p* < 0.05. **, *p* < 0.01. ***, *p* < 0.001.).

### Measurement of ROS generation

ROS levels in *G. lamblia* trophozoites were determined as previously described [108, 109]. WB clone C6 trophozoites were cultured in growth medium with indicated drugs and with respective controls for 24 h. About 2 × 10^6^ cells were harvested, washed in PBS, and incubated with 2’, 7’-dichlorodihydrofluorescein diacetate at a concentration of 25 μM in 200 μl cell suspensions. The cell suspensions were kept in dark at room temperature for 1 h. Data were collected from a Paradigm Multi-Mode Plate Reader (Beckman Coulter) at 530 nm after excitation at 488 nm.

### Co-Immunoprecipitation assay

The stable cell lines were cultured in growth medium or inoculated into encystation medium with puromycin and harvested and lysed after 24 h as previously described [103]. The cell lysates were incubated with anti-HA antibody conjugated to beads as previously described [103]. For reciprocal immunoprecipitation experiments, anti-MLF was used to do immunoprecipitation. The lysates were incubated with 2 μg of anti-MLF antibody or preimmune serum for 2 h and then incubated with protein G plus/protein A-agarose (Merck) for 1 h. Proteins from the beads were analyzed by Western blotting using anti-HA monoclonal antibody (1/5000 in blocking buffer; Sigma), anti-MLF (1/10000 in blocking buffer)[47], or anti-RAN (1/10,000 in blocking buffer)[106], as previously described [103].

## Supplementary Information

**Additional file 1**. Supplementary figures and tables.

## Data Availability

Not applicable.

## Abbreviations

CWPs: Cyst wall proteins
MLF: Myeloid leukemia factor
MLFVs: MLF vesicles
CDK2: Cyclin-dependent kinase 2
*neo*: Neomycin phosphotransferase
ROS: Reactive oxygen species
ATG8L: ATG8-like
KD: Knockdown
