# Supplementary material for: A myeloid leukemia factor homolog is involved in tolerance to stresses and stress-induced protein metabolism in Giardia lamblia
Source: Biol Direct. 2023 Apr 24;18:20. doi: 10.1186/s13062-023-00378-6 (PMC10127389; doi:10.1186/s13062-023-00378-6)
Supplement: Supplementary file 1 — Additional file 1. Supplementary figures and tables. [file 13062_2023_378_MOESM1_ESM.pdf]

Fig. S1

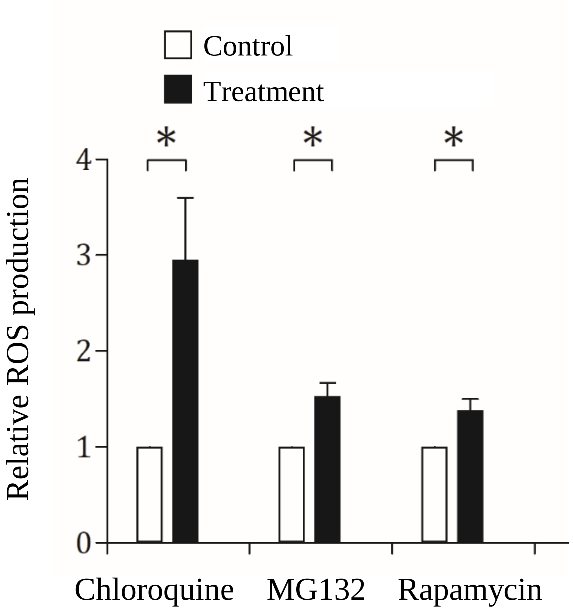

Fig. S1. Chloroquine, MG132, and rapamycin treatment increased ROS production. The wild-type non-transfected WB cells were cultured in growth medium with 100μM chloroquine, 80μM MG132, and 36μM rapamycin, or the same volume of solvent (H2O or Me2SO) for 24 h and then subjected to ROS measurement as described under “Materials and Methods”. Fold change is calculated as the ratio of the difference between the treatment group and control group, to which a value of 1 was assigned.

Fig. S2

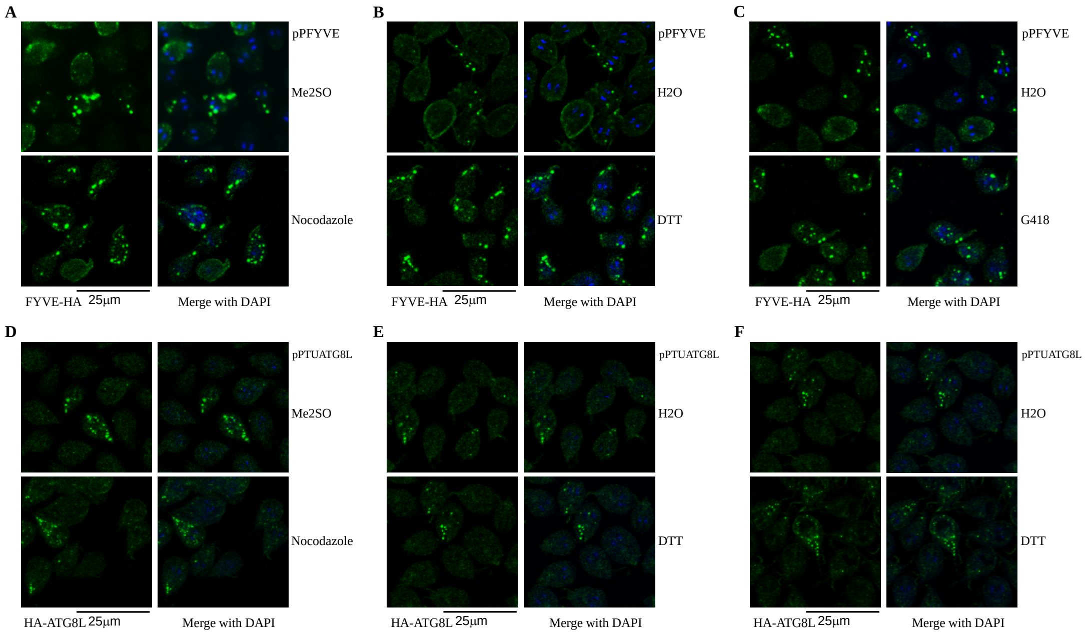

Fig. S2. Nocodazole, DTT, and G418 treatment increased the number of FYVE- and ATG8L-localized vesicles. (A)(B)(C) FYVE-localized vesicles can be induced by nocodazole, DTT, and G418 treatment. The pPFYVE stable transfectants were cultured in growth medium with 5μM nocodazole, 5mM DTT, and 217μM G418, or the same volume of solvent (H2O or Me2SO) for 24 h and then subjected to immunofluorescence assay using anti-HA antibody for detection.(D)(E)(F) ATG8L-localized vesicles can be induced by nocodazole,DTT, and G418 treatment. The pPTUATG8L stable transfectants were cultured in growth medium with 5μM nocodazole, 5mM DTT, and 217μM G418, or the same volume of solvent (H2O or Me2SO) for 24 h and then subjected to immunofluorescence assay using anti-HA antibody for detection.

Fig. S3

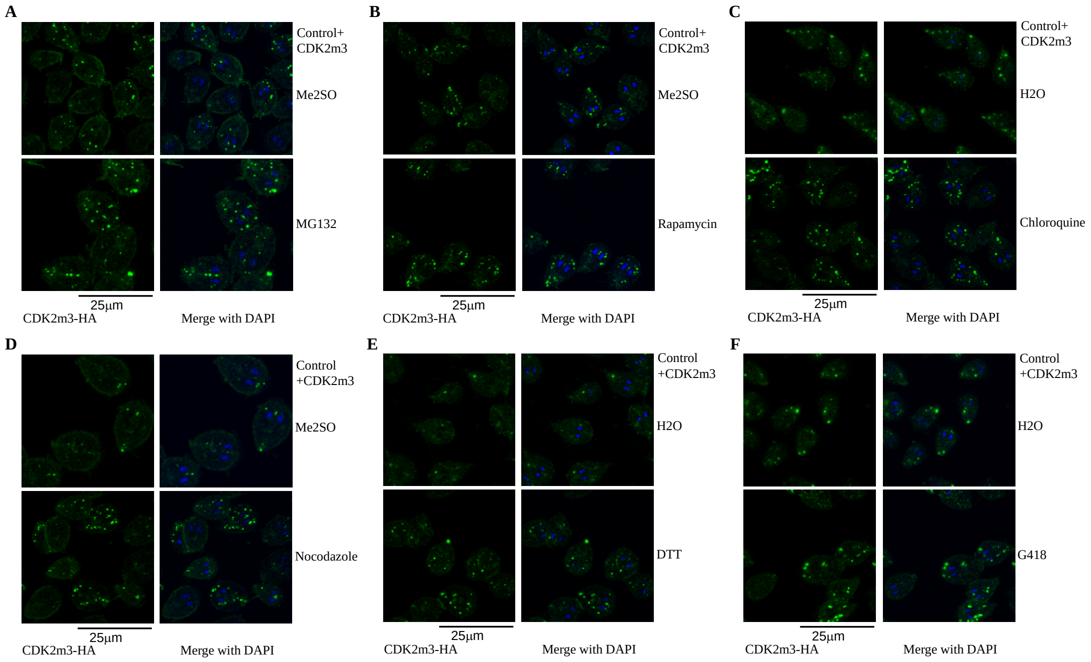

Fig. S3. MG132, rapamycin, chloroquine, nocodazole, DTT, and G418 treatment increased the number of CDK2m3-localized vesicles. The control+CDK2m3 stable transfectants were cultured in growth medium containing (A) 80μM MG132, (B) 36μM rapamycin, (C) 100μM chloroquine, (D) 5μM nocodazole, (E) 5mM DTT, and (F) 217μM G418, or the same volume of solvent (H2O or Me2SO) for 24 h and then subjected to immunofluorescence analysis using anti-HA antibody for detection.

Fig. S4

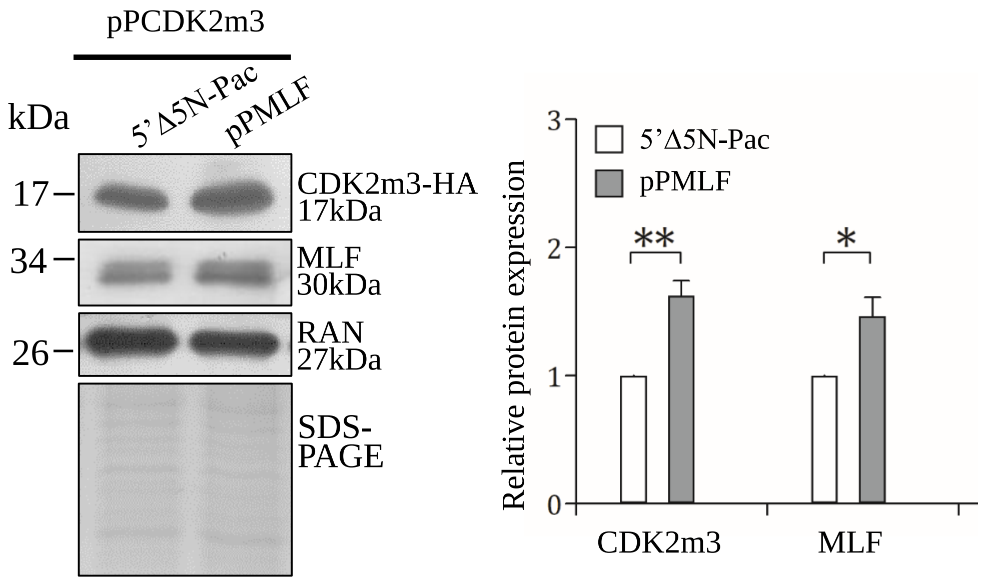

Fig. S4. Transfection of MLF expression vector increased CDK2m3 protein level. Transient co-transfection of pPCDK2m3 transfectants with 5'Δ5N-Pac or pPMLF plasmid was performed. At 24 h after Transfection, the cell lines cultured in growth medium were subjected to SDS-PAGE and Western blot analysis using anti-HA, anti-MLF, and anti-RAN antibodies, respectively. SDS-PAGE with Coomassie Blue staining is included as a control for equal protein loading. The band intensity from triplicate Western blots was quantified using Image J as described in Fig. 1E. *, $p < 0.05$. **, $p < 0.01$.

Table S5. Oligonucleotides used for construction of plasmids and PCR.

Name	Sequence (5'--->3')
18SrealF	AAGACCGCCTCTGTCAATCAA
18SrealR	GTTTACGGCCGGGAATACG
mlfrealF	AGCATTTTTGGAAACATGGAT
mlfrealR	AAGCAGCGCATAGTCTCAAAC
cwp1realF	AACGCTCTCACAGGCTCCAT
cwp1realR	AGGTGGAGCTCCTTGAGAAATTG
cwp2realF	TAGGCTGCTTCCCACTTTTGAG
cwp2realR	CGGGCCCGCAAGGT
ranrealF	TCGTCCTCGTCGGAAACAA
ranrealR	AACTGTCTGGGTGCGGATCT
neomF	TCTCGTCGTGACCCAcGGCGATGCCTGCTTG, the mutated sequence in the neo gene is shown in lower case
neoXR	GGCCGCTCGAGTCAGAAGAACTCGTCAAGAAG
neomR	CAAGCAGGCATCGCCgTGGGTCACGACGAGA, the mutated sequence in the neo gene is shown in lower case
neoNF	GGCCGCCATGGATGATTGAACAAGATGGATTG
hMLF2F	CACCATGTTCCGCTTCATGAGGGACGTG
hMLF2R	CCAGTCATAGCGGCGGGACTG
MLFNF	GGCGGCTAGCTGTGTAAGCAACACGTAGTTC
MLFMR	GGCGACGCGTGTAGCGACGATTACCGGA
mlfmF	CATACGAGAGCAagcagccacgagTACATATCGAATgagggtattgggcacagcggaccgagtggtGAAGACGTGAGT, the mutated region in the *mlf* gene is shown in lower case, the encoding peptide changes from "IGDKYISNKRERDIRTGR"to "SSHEYISNEGIGHSGPSG"
mlfmR	ACTCACGTCTTCACCACTCGGTCCGCTGTGCCCAATACCCTCATTCGATATGTACTCGTGGCTGCTTGCTCTCGTATG, reverse sequence of mlfmF

hMLF2 fragment	GGCCAT**GCTAGC**aattgaagcttaaagatgacgaaagcctgtagcccagtagtaaataccccaagatttgtctgattgacaaattgattctgaatggttgctgaggattgtgattgacatacgtttacttacaagcggaagtcttattgtagccatgtatgtagatgctacgaaggtctaaatcaattccgaatcaactatactaacctatcatgatgaccatgatgcaacgcaaaaaataaaaATGTTCCGCTTCATGAGGGACGTGGAGCCTGAGGATCCCATGTTCCTGATGGATCCCTTTGCTATTCACCGTCAGCATATGAGCCGTATGTTGTCAGGTGGCTTTGGATATAGCCCCTTCCTCAGCATCACAGATGGCAACATGCCAGGGACCAGGCCTGCCAGCCGCCGGATGCAGCAGGCTGGAGCTGTCTCCCCCTTTGGGATGCTGGGAATGTCGGGTGGTTTCATGGACATGTTTGGGATGATGAATGACATGATTGGAAACATGGAACACATGACAGCTGGAGGCAATTGCCAGACCTTCTCATCTTCCACTGTCATCTCCTACTCCAATACGGGTGATGGTGCCCCCAAGGTCTACCAAGAGACATCAGAGATGCGCTCGGCACCAGGCGGGATCCGGGAGACACGGAGGACTGTTCGGGATTCAGACAGTGGACTGGAGCAGATGTCCATTGGGCATCACATCCGGGACAGGGCTCACATCCTCCAGCGCTCCCGAAACCATCGCACGGGGGACCAGGAGGAGCGGCAGGACTATATCAACCTGGATGAGAGTGAGGCCGCAGCGTTTGATGACGAGTGGCGGCGGGAGACCTCCCGATTCCGGCAGCAGCGTCCCCTGGAGTTTCGGCGGCTTGAGTCCTCAGGGGCTGGGGGACGAAGGGCGGAGGGGCCTCCCCGCCTGGCCATCCAGGGACCTGAGGACTCCCCTTCCCGACAGTCCCGCCGCTATGACTGG**ACGCGT**AGGCTG, the 5’ untranslated region of *Giardia mlf* gene is shown in lower case, the bold region is the NheI and MluI sites, the underlined region is the *hmlf2* gene
